# More Doctors, better health? A generalised synthetic control approach to estimating impacts of increasing doctors under Brazil’s Mais Medicos Programme

**DOI:** 10.1016/j.socscimed.2024.117222

**Published:** 2024-08-17

**Authors:** Rhys Llewellyn Thomas, Christopher Millett, Ricardo de Sousa Soares, Thomas Hone

**Affiliations:** 1 Health Economics Research Centre, Nuffield Department of Population Health, University of Oxford, Oxford, UK; 2 Public Health Policy Evaluation Unit, Imperial College London, London, UK; 3 NOVA National School of Public Health, Public Health Research Centre, Comprehensive Health Research Center, CHRC, NOVA University Lisbon, Lisbon, Portugal; 4 Department of Health Promotion, Federal University of Paraíba, João Pessoa, PB, Brazil

**Keywords:** Synthetic Control, Brazil, Human resources for health, Mortality, Hospitalisations, Primary care, Doctors

## Abstract

Worldwide, there are an insufficient number of primary care physicians to provide accessible, high-quality primary care services. Better knowledge on the health impacts of policies aimed at improving access to primary care physicians is important for informing future policies.

Using a generalised synthetic control estimator (GSC), we estimate the effect of the increase in primary care physicians from the *Programa Mais Médicos* in Brazil. The GSC allows us to estimates a continuous treatment effects which are heterogenous by region. We exploit the variation in physicians allocated to each Brazilian microregion to identify the impact of an increasing *Mais Médicos* primary care physicians. We explore hospitalisations and mortality rates (both total and from ambulatory care sensitive conditions) as outcomes. Our analysis differs from previous work by estimating the impact of the increase in physician numbers, as opposed to the overall impact of programme participation. We examine the impact on hospitalisations and mortality rates and employ a panel dataset with monthly observations of all Brazilian microregion over the period 2008-2017.

We find limited effects of an increase in primary care physicians impacting health outcomes - with no significant impact of the *Programa Mais Médicos* on hospitalisations or mortality rates. Potential explanations include substitution of other health professionals, impacts materialising over the longer-term, and poor within-region allocation of *Mais Médicos* physicians.

## Introduction

Strengthening primary healthcare is essential for building accessible and high-quality health systems. Investments in primary healthcare can increase equity and access, raise service quality, foster system accountability, and improve health outcomes” (WHO, 2021). Strong primary care systems have been linked to better health outcomes, lower costs, fewer unnecessary hospitalisations (Guanais & Macinko, 2009; Macinko et al., 2010; Rao & Pilot, 2014; Rasella et al., 2014; Starfield et al., 2005; Starfield & Shi, 2002), and reductions in health inequalities (Hone et al., 2017; OECD, 2020; Szwarcwald et al., 2010). Appropriately distributed, motivated, and trained primary care physicians are an essential component of high-quality primary care. However, in 2006 it was estimated that there was a deficit of 2.4 million physicians, nurses and midwives worldwide (WHO, 2006), with major implications for service delivery.

Insufficient primary care physician numbers remain a global concern. It has been found that over 14,000 primary care physicians are required to reduce the deficit in the US (The Kaiser Family Foundation, 2020) and this deficit is estimated rise to 40,400 by 2036 (GlobalData Plc., 2024). A lack of primary care physicians is not limited to the US. In Canada, 4.6 million people do not have access to a regular primary care physician (Kiran, 2022), and in the United Kingdom there is expected to be a shortfall of 8,800 primary care physicians by 2030 (The Kaiser Family Foundation, 2020). These shortages have also been found to be present in Germany (van den Bussche, 2019), and France (Ministère du Travail, de la Santé et des Solidarités, 2022), indeed it is claimed that 31% of the central Europe, eastern Europe, and central Asia group of countries have physician shortages (Haakenstad et al., 2022).

These physician shortages have significant implications for service delivery, including worsening access to healthcare services, increased hospitalisations (Kravet et al., 2008), greater use of emergency care services, and rising healthcare costs (Shi, 2012). Physician shortages also likely impact the time physicians can spend with patients, which is a barrier to providing quality care (Deshpande & DeMello, 2011). These impacts also have downstream effects on patients’ health. Evidence from the US indicates that an additional 10 primary care physicians per 100,000 people is associated with a 98-day increase in life expectancy (Basu et al., 2019), and higher physician density is also associated with lower all-cause mortality (Macinko et al., 2007).

Physician shortages are particularly acute in many low- and middle-income countries (LMICs) (Hoyler et al., 2014; WHO, 2006). Sub-Saharan Africa, some Asian countries and Latin America have particularly low physician densities by global standards (Haakenstad et al., 2022). However, national figures mask maldistribution within countries, and there are regions within these countries that face even more serious shortages, especially in rural regions (OECD, 2019; Viktor et al., 2021). Physician deficits stem from poor working conditions, insufficient medical training, and workforce migration (Campbell et al., 2013; Chen et al., 2004). While in rural regions the proposed causes of shortages are the relatively worse living conditions, work-life balance, and professional support (Adynski & Morgan, 2021; Kumar & Clancy, 2021).

Brazil is middle income country with challenges in appropriate distribution of health professionals across geographical regions and between the public and private sectors. Physician numbers in Brazil are low compared to similar countries. In 2019 the average physician density in Brazil was 2.3 per 1,000 population whereas the average for South America was 2.4 per 1,000 (The World Health Organisation, 2022). There is also significant regional variation in physician densities, with the poorest states having as few as 0.71 physicians per 1000 population, and the richest having 4.09 per 1000 population (Santos et al., 2017). The especially low figure for poorer regions are comparable in magnitude to countries of sub-Saharan Africa, which had an average of 1.0 physicians per 1000 in 2004 (Chen et al., 2004).

To alleviate these physician shortages, the Brazilian government launched the *Programa Mais Médicos* (PMM), which aimed to improve the provision of primary care to underserved communities, by increasing primary care physician supply (Mattos & Mazetto, 2019). Initially the PMM aimed to train more physicians and redistribute already trained physicians to underserved areas of Brazil. However, the initial take-up of the programme by Brazilian physicians was below expectations. To make up or this shortfall the Brazilian government signed an agreement with Cuba, negotiated by the Pan America Health Organisation (PAHO), to allow Cuban doctors to fill PMM vacancies in primary care (Harris & Harris, 2016; Mattos & Mazetto, 2019; Pereira et al., 2016; Santos et al., 2017). Over 14,000 Cuban physicians came to Brazil to fill these PMM vacancies. Alongside the increase in physician numbers, the programme also aimed to establish new medical schools, and to provide funding for construction and refurbishment of clinics (Hone et al., 2020).

Studies have found significant increases in physician numbers, and increased number of consultations due to the PMM (Carrillo & Feres, 2019; Hone et al., 2020; Pereira et al., 2016). However, studies analysing the effectiveness of the PMM have found limited evidence of an impact on various health outcomes, including infant health outcomes (Bexson et al., 2021; Carrillo & Feres, 2019), mortality (Mattos & Mazetto, 2019), hospitalisations (Mattos & Mazetto, 2019), and ACSC hospitalisations (Fontes et al., 2018). Some studies have found very small reductions in amendable mortality and infant mortality associated with the PMM, but only in areas with the highest health burdens and fewest doctors pre-PMM (Bexson et al., 2021; Hone et al., 2020) – thus suggesting that if doctors had been allocated only to areas with low doctor numbers there may have been a larger overall effect.

The PMM literature has focused on examining the impacts of the program as a binary variable, focusing on participation in the programme rather than using the number of additional doctors each region received. Previous studies have provided rigorous estimates of the effects of programme participation, but they have not considered the substantial variations in physician numbers across regions. This study seeks to estimate the health impacts of increased doctor density by using the variation in the number of physicians that regions received through the PMM.

This paper investigates the impact of the PMM on health outcomes by modelling increases in physician density—like a dose-response— rather than participation in the programme. We also investigate the impact of non-PMM physicians on the same outcomes. Our analysis uses a balanced panel of microregions from 2008 to 2017 and a Generalised Synthetic Control (GSC) estimator. This method offers advantages over the difference-in-differences (DiD) estimators in our context. Specifically, the GSC estimator calculates a continuous average treatment effect, such an increase in PMM physician density, without requiring the "strong parallel trends" assumption needed for continuous DiD estimators. As the name suggests, this assumption is a stricter form of the parallel trends assumption (Callaway et al., 2024). The strong parallel trends assumption required for the continuous DiD imposes constraints on the treatment effect heterogeneity, while the GSC approach does not require constant treatment effects across regions – i.e. increases in physicians had identical impacts in each region that received PMM physicians. Given the size and diversity of Brazilian regions, it is unlikely that effects of additional doctors are constant across regions, and would therefore leads to biased estimates (Athey & Imbens, 2018; Borusyak & Jaravel, 2017; Callaway & Sant’Anna, 2020; de Chaisemartin & D’Haultfoeuille, 2021; de Chaisemartin & D’Haultfœuille, 2020; Sun & Abraham, 2020).

Our contribution to the literature is threefold: Firstly, we provide evidence of the impact of an additional PMM doctor on hospitalisations and mortality as a marginal treatment effect. This adds to the existing literature on the PMM programme, which has primarily focused on the impact of participation in the programme. Secondly, our paper contributes estimates of the impact of a unit increase primary care doctor density in a middle-income country, showing that, in contrast to the findings of (Nikoloski et al., 2021), there is a limited effect of increasing primary care physician density on hospitalisation or mortality rates. Lastly, we present evidence which suggests regional variation in these effects. Our tentative evidence indicates that the largest decreases in mortality occur in areas with larger rural populations, lower nurse densities, and higher poverty levels. These results are useful for ensuring effective allocation of doctors in the new *Programa Mais Medicos*, and similar programmes which aim to improve public health through the expansion of primary care physician supply.

### *Programa Mais Médicos* (PMM) Background

In July 2013 Programa Mais Médicos (PMM) was launched in Brazil with one of the main objectives being to reduce primary care physician deficits in underserved areas (Mattos & Mazetto, 2019). The programme comprised of three main components: to establish new medical schools, provide funding for construction and refurbishment of clinics, and an emergency increase in the number of primary care physicians. This was achieved, firstly, by offering generous salaries, as well as housing and food benefits, to Brazilian physicians that relocated to work in areas with shortages of physicians. However, only 10% of the vacancies were filled in the first round (Pereira et al., 2016). To fill the remaining vacancies, an international cooperation was made between Brazil and Cuba, facilitated by Pan American Health Organization (PAHO), which allowed the participation of Cuban physicians in the PMM (Harris & Harris, 2016; Santos et al., 2017). For context, the physician density in Cuba is high by international comparison, with a density of 8.4 per 1,000 population in 2018, which is the highest of any country in the Americas (including Canada and the US) (The World Health Organisation, 2022). PMM physicians from abroad were required to participate in additional training, including Portuguese language classes and background on Brazil and the healthcare system (Mattos & Mazetto, 2019).

The Ministry for Health in Brazil set a criteria for prioritising allocation of the PMM doctors to municipalities. Municipalities were considered a priority municipality for receiving PMM physicians if: 20% or more of the population were in extreme poverty; they were one of the 100 municipalities with the lowest income per capita and a population over 80,000; they were state capitals, metropolitan regions or municipalities with extreme poverty; or if they had a low human development index, or were semi-arid with Quilombo communities (Carrillo & Feres, 2019; Hone et al., Ministério da Saúde, 2018; 2020; Oliveira et al., 2016).

For a municipality to receive PMM physicians they were required to express their interest in participating in the programme. However, the priority criteria was not strictly adhered to and many non-priority municipalities received physicians under the programme (Oliveira et al., 2016). By 2018, the programme was implemented in 4,524 of the 5,570 municipalities. As a result of critical comments by ex-President Jair Bolsonaro, the Cuban government withdrew Cuban physicians from Brazil in November 2018, which brought an end to Cuban physicians involvement in the programme (Santos et al., 2018). In 2023, with the election of Lula, the PMM is once again a health policy priority. Since Lula’s election there have been changes in legislation for filling vacancies and new incentives for the provision of doctors in the country (MEDIDA PROVISÓRIA N° 1.165, DE 20 DE MARÇO DE 2023 - DOU - Imprensa Nacional, 2023).

Appendix Figure 11 shows PMM and non-PMM primary care physician density by microregion. This figure shows the substantial variation in physician density by microregion, and the very low density in the North-West of Brazil. The figure also shows that there is large variation in the number of PMM physicians each region received. Finally, it also shows a pattern, in that regions with lower density pre-PMM disproportionately received more PMM physicians than regions with already high primary care physician density.

## Methods

This paper estimates the impact of an increase in physician density, both PMM and non-PMM doctors per 100,000 population, on hospitalisation and mortality rates (per 100,000 population). The analysis uses a balanced panel dataset of Brazilian microregions over the period 2008–2017 and a GSC methodology. The GSC methodology is a powerful empirical tool for assessing the role programme intensity played on health outcomes (O’Neill et al., 2020; Powell, 2021).

### Data

We construct a panel of microregions with monthly observations for the years 2008 to 2017. Microregions were, until 2017, one of five geographic levels which divided Brazil. There are 26 States (and Distrito Federal) in Brazil, and these could be divided into 136 mesoregions. Mesoregions were groups of microregions (558 in total), which themselves were comprised of municipalities (5,570). Further detail on the number of microregions in each state, and the average population size of those microregions are available in Appendix Table 1. Only data from 2008 onwards is used due to substantial changes in primary care in 2006 (Paim et al., 2011). We seek to avoid including structural breaks to generate robust synthetic controls for each microregion.

Data was compiled from several sources. The Brazilian Ministry of Health website provided information on mortality, physicians, hospital beds, private health insurance plans, and municipal health expenditures. Additionally, we obtained data on the number of PMM physicians from the Brazilian Ministry of Health. The 2010 Brazilian Census provided demographic and socio-economic data for municipalities. For geographic data used in our figures, we consulted the Brazilian National Institute of Geography and Statistics (Instituto Brasileiro de Geografia e Estatistica, 2020).

### Outcomes

Our outcomes of interest are total hospitalisations, total mortality, and ACSC (Ambulatory Care Sensitive Condition) mortalities and hospitalisations (per 100,000 population) (Hodgson et al., 2019; Purdy et al., 2009; Sundmacher et al., 2015). The definition of ACSC is based on the Brazilian list (Ordinance number 221, 17 April 2008, Ministry of Health, Brazil), which includes nutritional diseases and anaemia, asthma and pulmonary diseases, hypertension, heart failure, cerebrovascular diseases, diabetes, epilepsy, and gastric ulcers. We calculated these rates by aggregating the municipality-level counts of the outcomes of interest to the microregion-level and dividing by the microregion population, which was calculated by summing the municipality-level populations from the 2010 census.

### Exposure

The exposure (or treatment) variables analysed in this study are the density of public primary care physicians per 100,000 population. Primary care doctors were defined based on whether they worked in a primary care facility, not by their speciality. We consulted the Brazilian Ministry of Health website for data on physician numbers and obtained data on PMM physicians from the Brazilian Ministry of Health. We did not access any specific information on the type of contract they held, or whether they relocated. We separately analysed PMM physicians and non-PMM physicians, determining the numbers of each based on their contracts with the Ministry of Health. We normalised physician numbers to full-time equivalents (FTEs), based on a 40-hour working week, using their reported employment hours. Primary care physicians are those reporting their ambulatory working hours in primary care facilities (health centres and posts, family health units, mixed healthcare units, water-based clinics serving fluvial communities, and indigenous health centres).

As for hospitalisation and mortality rates, we calculated per 100,000 density of physicians by dividing the number (municipality numbers aggregated to the microregion-level) by the microregion population (municipality population data from the 2010 Census aggregated to the microregion-level).

### Microregion Characteristics

In additional analysis, we used microregion characteristics. Specifically, we used data from the 2010 Brazilian Census on the percentage of extremely poor individuals, the Human Development Index, and the percentage of the rural population. We aggregated these to the microregion level from municipality-level data by taking a population-weighted average of the municipality-level observations. Additionally, we accessed data on health facilities and nurses from the Brazilian Ministry of Health website, converting these to densities (per 100,000 population) using the same procedures described for physician density. For the post estimation correlation analysis, we use the 2012 average for health facilities per 100,000 population, nurses per 100,000 population, and density of primary care physicians.

### Generalised Synthetic Control

In our analysis, we use the GSC estimator developed by (Powell, 2021). The GSC exploits variation over time and between microregions, modelling differences in physician densities and their impacts on hospitalisations and mortality rates. The GSC is an extension of the traditional synthetic control estimator, which is typically uses one treated unit and several untreated units. While the traditional approach uses a selection of untreated units to estimate a counterfactual unit, the GSC extends this by using all available units as potential donors, which allows for a continuous treatment effect to be estimated. Unlike traditional difference-in-differences estimators, which rely on a pre-treatment period and a set of control units, the GSC estimates the impact of physician density by creating a control unit for each microregion using a combination of other microregions, this control unit is called the synthetic control. This synthetic control is generated so that its outcomes evolve in a similar way over time. One benefit of the GSC is that it does not assume constant treatment effects, thus allowing for microregion-specific effects to be estimated which might arise from factors such as initial physician numbers, whether the region is urban or rural, or the number of physicians the municipality received. The GSC methodology also permits correlations between the number of PMM physicians received and specific characteristics of microregions, which would usually cause selection bias.

The GSC models outcomes as:

$$\begin{array}{c} Y_{i,t}^{N}=\lambda_{t}\mu_{i}+\epsilon_{i,t}\\ Y_{i,t}=Y_{i,t}^{N}+D_{i,t}^{\prime }\alpha_{i} \end{array}$$

Where *Y*_*i*,*t*_ is the outcome (hopsitalisation or mortality rate) for state *i* at time *t*, $Y_{i,t}^{N}$ is the un-treated outcome, which requires estimation. *D*_*i*,*t*_ is the treatment variable (PMM doctor density), *λ*_*t*_ is a vector of common unobserved factors, and *μ*_*i*_ are the factor loadings. *α*_*i*_ is the microregion-specific estimate of the effect, which is also estimated. The un-treated outcome $Y_{i,t}^{N}$ is estimated by taking a weighted combination of untreated donor units $Y_{j,t}^{N}$:

$$Y_{i,t}^{N}=\sum_{i\neq j}^{N}w_{j}^{i}Y_{j,t}^{N}$$

Where $w_{j}^{i}$ is the weight given to donor *j* to create a synthetic control for microregion *i*. The GSC jointly estimates the weights to create the untreated synthetic control unit, and the micro-region specific estimate (*α*_*i*_).

As is shown, the GSC nests a two-way fixed effect model but allows for a more flexible outcome trajectories (Powell, 2021), accounting for trends and seasonality in the outcome by generating a synthetic control which also exabits these features. Indeed, the GSC methodology has previously been found to outperform difference-in-differences and interactive fixed effects methodologies in simulations (O’Neill et al., 2020; Powell, 2021).

An alternative approach to the GSC would be to use a continuous DiD (Callaway et al., 2024), but the GSC offers some advantages which are important in this context. To estimate an average treatment effect free of selection bias, a "strong parallel trends" assumption is necessary. This assumption does not allow units to select into a specific dose based on the expected benefits. It requires that if other microregions had received the same number of PMM physicians, then the changes in outcomes would have been the same as those observed in the microregions that actually received that number of PMM physicians. However, this seems unlikely in our context for two reasons. Firstly, the policy was designed to allocate PMM doctors to municipalities expected to benefit most. Secondly, variations in physician numbers prior to PMM roll-out suggest that receiving the same number of PMM physicians would not have had the same effects in different regions.

For the main analysis, the GSC method is used to estimate a different effect for each microregion. We generate the synthetic control for each microregion using only microregions in the same state to better approximate its characteristics (Abadie, 2021) and to reduce potential over-fitting issues. Using donor microregions within the same state ensures that the donor pool remains relatively small, which reduces the risk of biases due to overfitting. The states of Rondônia, Acre, Amazonas, and Roraima are combined as are the states of Pará, Amapá, and Tocantins, due to the relatively few microregions in those States. We present these microregion-specific effects graphically in appendix figures 15-18 and we aggregate these effects to the state-level by taking a weighted average of the microregion-specific effects. Microregions with better fitting Synthetic Controls are given additional weight (Powell, 2021).

We use microregion-level data, despite having some municipal-level data, due to issues associated with over-fitting and noise (i.e., random variation in the outcome variables). In choosing the level of geographic aggregation, there is a bias-variance trade-off. Although aggregating to the microregion level may average out important heterogeneity within microregions that could bias our results, the data from larger geographic regions will be less noisy, as random variation from smaller geographic regions would be smoothed out. Additionally, having many donors but relatively few time periods can introduce biases in the estimates through over-fitting (Abadie, 2021). Therefore, aggregating to the microregion level has the added benefit of reducing the number of donors. For these reasons, microregions are the most suitable geographical unit for this analysis.

### Post Estimation Correlations

We explore the relation between microregion effect estimates and their socioeconomic and demographic characteristics. We present correlations from a bootstrapped Pearson’s correlation coefficient, and a Spearman correlation coefficient to reduce the influence of outliers. We use correlations without statistical testing because traditional statistical tests do not consider the uncertainty in the value of the effect estimate. We take the microregion-specific effect estimated using the GSC procedure, and present graphical analysis of their association with microregion characteristics: percentage of extremely poor, Human Development Index, percentage rural population, health facilities per 100,000 population, nurses per 100,000 population, and pre-PMM density of primary care physicians (per 100,000 population).

### GSC Assumptions

To claim causality, several key assumptions are required. Firstly, a good synthetic control for each microregion and graphical comparison of the pre-treatment outcomes of the synthetic control and the true outcomes is necessary. In the appendix of this paper, we present the average Root Mean Squared Error (RMSE) of the synthetic microregion and the true outcome for that microregion, separately for each state over the period pre-PMM roll-out. Appendix Figure 1 presents these for total hospitalisations, while Appendix Figure 2 presents them for total mortality. These figures show that there is some variation over time in the RMSE for each state, however there is no clear pattern in the RMSE. Overall, the RMSE are also relatively small (approximately 10% of the outcome for mortality, and for hospitalisations is about 8.1% of the outcome). We also present figures with the average outcome and average synthetic control together in appendix Figures 3 and 4. Overall this evidence suggests that reasonable counterfactuals are estimated for each microregion.

We also need to assume that there is no anticipation of physician density. It is theoretically unlikely that mortality or hospitalisation would change in the expectation of receiving more primary care physicians in the future. To test whether future physician density impacts current mortality or hospitalisations we estimate the GSC for each state, as we do in the main paper, but instead using 6-months’ worth of leads for our treatment variable. We test leads of up to 6 months, separately for each state, and by PMM and non-PMM physicians, and find no evidence of anticipation. A further discussion of this assumptions and the tests we conduct is available in the appendix of this paper. Finally, we require no interference between states - i.e. no spillover effects. This may be violated if individuals cross microregion borders to access treatment, or if physician move across microregion borders to provide care, but this is unlikely to be the case due to the rurality of many of the areas under study. Because we are unable to test this assumption and therefore definitively rule out the existence of interference, this may be considered a reasonable limitation of our work.

## Results

### Descriptive Statistics

A total of 558 microregions were included in the analyses between 2008 and 2017. Prior to 2013 the total number of physicians working in primary care was stable at 61,000 but increased to approximately 68,000 during 2015 (Appendix Figure 4). From July 2013 there was a gradual increase in the number of PMM physicians eventually stabilising at 17,000 in 2015. Because some non-PMM doctors became PMM doctors, the overall increases in primary care doctors was around 6,000.

Prior to the PMM roll-out, the average hopsitalisation rate (per 100,000 population) of a microregion was 537.5, whilst the average ACSC hospitalisation rate was 143.0. For mortality the microregion average was 48.23 per 100,000 and the ACSC equivalent was 14.7. Appendix Figure 5 show the raw values for total and ACSC hospitalisation over our study period, and Appendix Figure 6 show the total and ACSC mortality. Hospitalisations remained relatively stable across our study period, whilst there were marginal increases in mortality over the course of the study period and increases from approximately 87,000 total deaths in January 2008 to 107,800 by the end of 2018.

### GSC results

**Figure 1:**
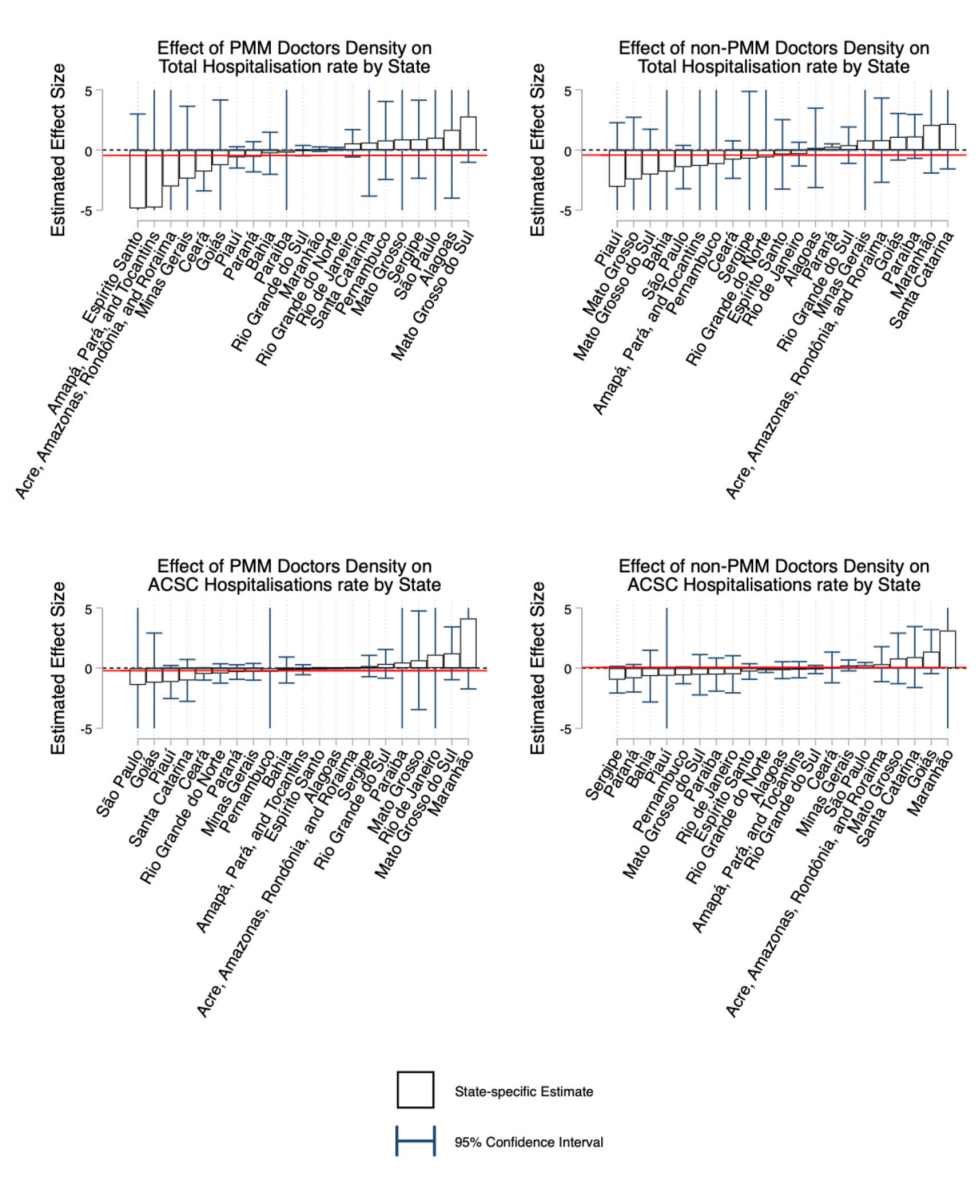
Effect of physician density on total hospitalisation rate using the generalised synthetic control estimator Figure present the average marginal treatment effects for each state estimated using the GSC, and ordered by magnitude. Blue bars represent 95% confidence intervals. These confidence intervals are calculated by using the inference procedure outlined by Powell (2021) and using those p-values to calculate the corresponding t-statistic, standard error and subsequently confidence intervals. Bars without caps have confidence intervals outside of the range of the graph. The red line represents the population weighted average estimate for the entire country.

**Figure 2:**
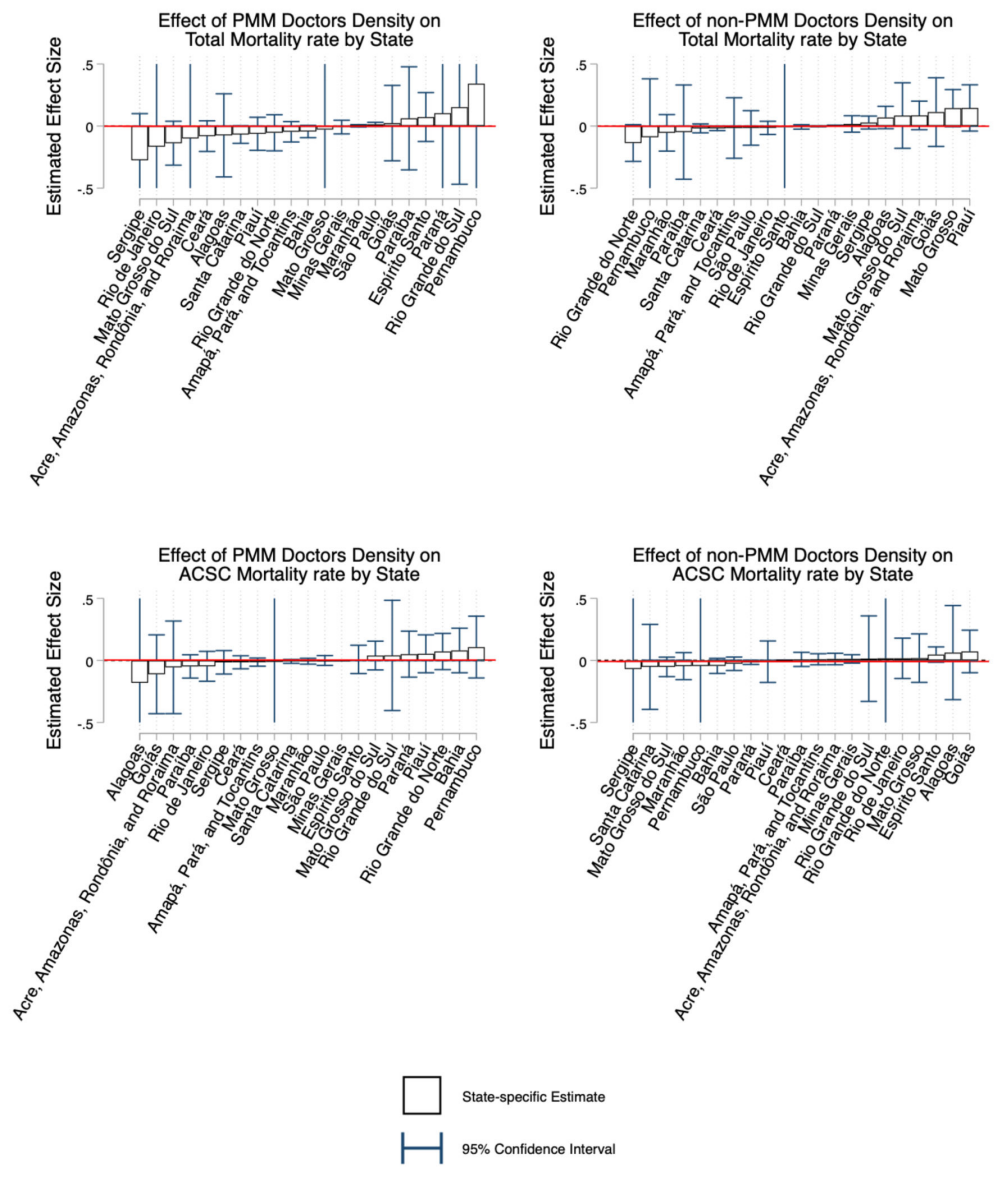
Effect of physician density on total mortality rate using the generalised synthetic control estimator Figure present the average marginal treatment effects for each state estimated using the GSC, and ordered by magnitude. Blue bars represent 95% confidence intervals. These confidence intervals are calculated by using the inference procedure outlined by Powell (2021) and using those p-values to calculate the corresponding t-statistic, standard error and subsequently confidence intervals. Bars without caps have confidence intervals outside of the range of the graph. The red line represents the population weighted average estimate for the entire country.

Figure 1 presents the GSC estimates of the effect of both PMM and non-PMM physician density on total and ACSC hospitalisation rate. Figure 2 presents the estimates for total mortality and ACSC mortality. These figures present the average marginal treatment effects for each state, ordered by magnitude. The bar represents the estimated effect, and the red line represents the population weighted average estimate for Brazil. In the appendix we also present each microregion-specific estimates graphically.

### Hospitalisation Estimates

In GSC models, there was no significant impact of changes in either PMM and non-PMM densities on hospitalisation rate either in total or from ACSCs. The country-level average impact (red line) is close to zero in all cases, suggesting no clear impact of PMM physicians on hospitalisations, on average. The estimates for PMM and non-PMM physicians are of a comparable magnitude, therefore there is no clear evidence that PMM physicians were less effective than non-PMM physicians. In Appendix figures 5 and 6 we show results from the joint estimation of PMM and non-PMM, which also show limited impacts on hospitalisation rates. Appendix figures 15 and 16 present the microregion specific estimates graphically.

### Mortality Estimates

There is no evidence of an impact of either PMM or non-PMM physicians on mortality. There are no states which have significant estimates for any outcome, and the country average impact is very close to zero. These results suggest that on average primary care physicians had no impact on mortality of any type over this period. In Appendix figures 7 and 8 we show results from the joint estimation of PMM and non-PMM, which also show limited impacts on hospitalisation rates. Appendix figures 17 and 18 present the microregion specific estimates graphically.

### Post Estimation Correlations

**Figure 3:**
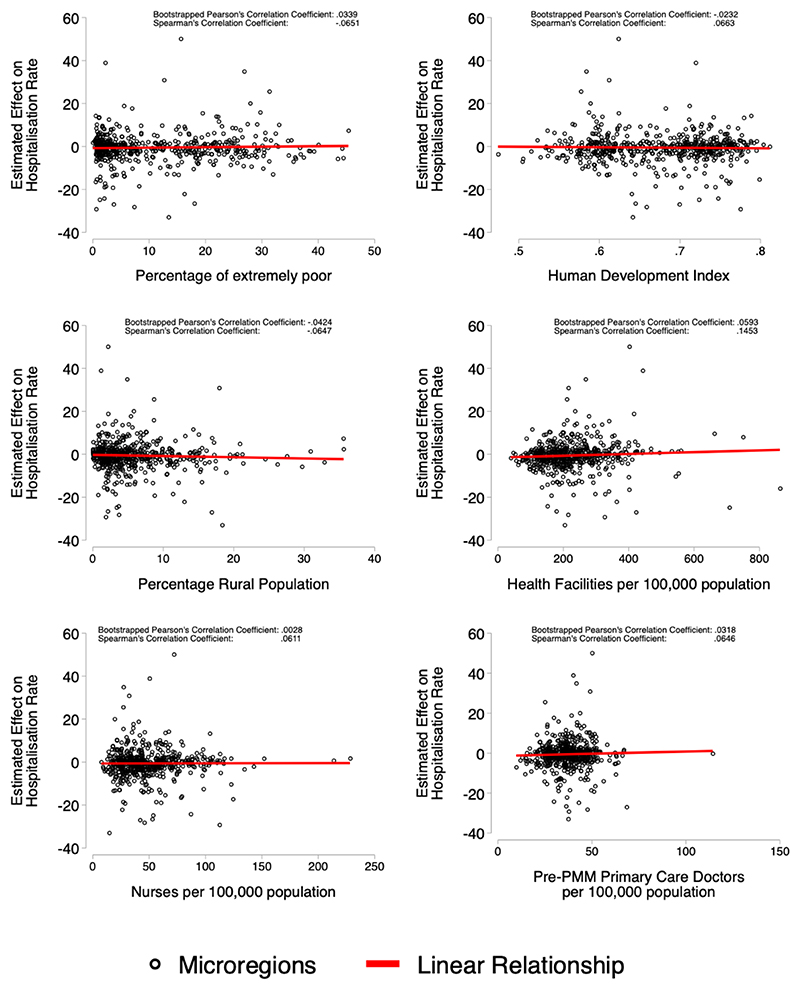
Correlation between Microregion effect size and Microregion Characteristics Figure shows the estimated microregion-specific effect on total hospitalisation rate – from the GSC procedure—on the y-axis and microregion characteristics on the x-axis. Two correlation coefficients are shown for each plot, bootstrapped Pearson’s correlation coefficient, and Spearman’s correlation coefficient.

**Figure 4:**
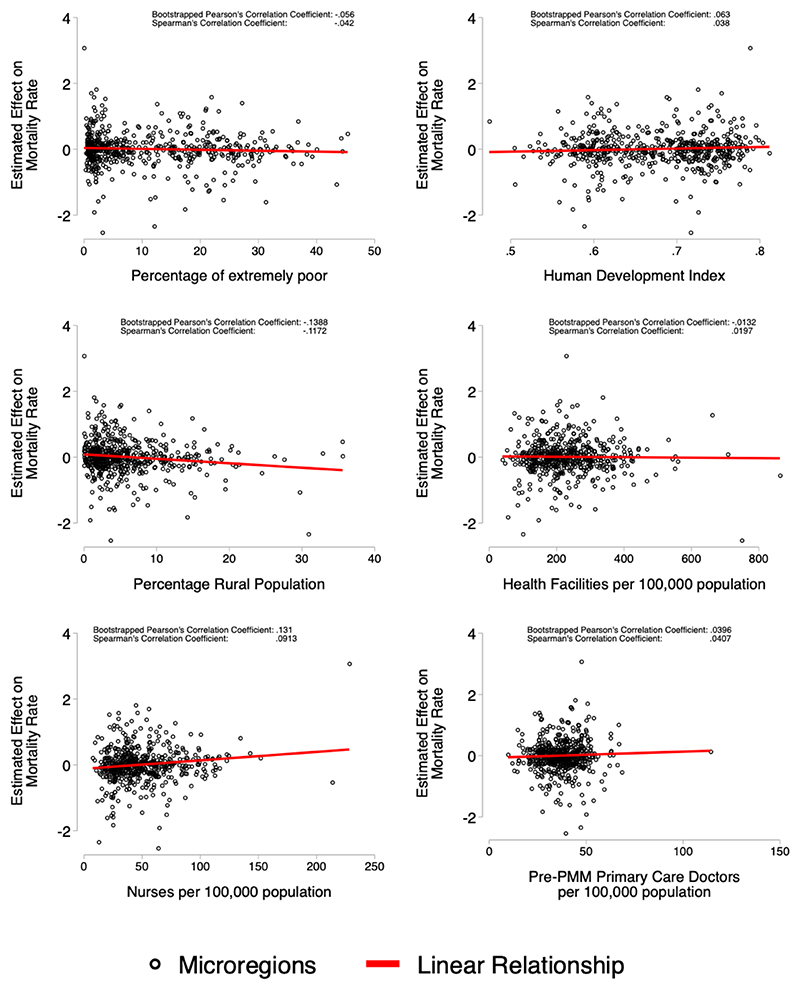
Correlation between Microregion effect size and Microregion Characteristics Figure shows the estimated microregion-specific effect on total mortality rate – from the GSC procedure—on the y-axis and microregion characteristics on the x-axis Two correlation coefficients are shown for each plot, bootstrapped Pearson’s correlation coefficient, and Spearman’s correlation coefficient.

Figures 3 and 4 show scatter plots, with the estimated effect for total hospitalisations or mortality on the y-axis and measures of microregion characteristics on the x-axis. Each circle represents a separate Brazilian microregion. There is very little association between microregion characteristics and estimates effect sizes for hospitalisation rates in Figure 3, while the correlations are somewhat stronger for mortality (Figure 4), they are still relatively small. The largest decreases in mortality associated with PMM physicians were from microregions which had larger rural populations, lower densities of nurses, and larger proportions of extremely poor.

## Discussion

This paper estimates the impact of PMM and non-PMM physician density on mortality and hopsitalisation rates, over the period 2008-2017. Using a novel GSC approach, the results of this study suggest that an increase in physician density through the PMM, on average, was not associated with changes in hospitalisation or mortality rates in any Brazilian state. There was some indication that poorer, rural areas with less developed health systems may have had associated reductions in mortality from the PMM, but our results do not conclusively find causal links between these factors and reductions in mortality.

These findings are generally in-line with those of previous work on the PMM (Carrillo & Feres, 2019; Mattos & Mazetto, 2019). Studies have found some evidence of reductions in amenable mortality associated with the PMM (Hone et al., 2020), however impacts were very small in magnitude, and concentrated in areas with low physician density pre-PMM. There are several explanations for these findings. Firstly, the timeframe we analyse is considered a short-term effect and we might expect the benefits of increasing physician density to materialise in the longer term, especially for mortality. The leading causes of mortality in our data were COPD (chronic obstructive pulmonary disease) and asthma, diabetes, and hypertension, and these conditions require long-term management and prevention. We explore whether the PMM physicians had any delayed effects in the Appendix, and find no evidence that they had any impact on hopsitalisaions or mortality even after 6 months. Secondly, evidence suggests physicians were not appropriately allocated to priority municipalities (Hone et al., 2020), and furthermore, it is possible that physicians were not allocated within priority municipalities to areas that most needed physicians. Our analysis suggest that an increase in nurse density was correlated with smaller impacts of physician increases on mortality rate –which is a similar finding to Carrillo and Feres (2019)—and may suggest areas with high nurse density were also not the most in-need of physicians. Thirdly, increases in primary care density may have had a limited impact on outcomes due to health system bottlenecks – for example geographic and financial barriers to access, and challenges with referral and access to secondary care healthcare system. Furthermore, it is important to consider the austerity policies implemented after 2016 in Brazil, and the impact on social inequalities, and on the reduction and turnover of doctors after the departure of Cubans and the changes in the provision of doctors in Brazil (Giovanella et al., 2019). These effects may have disproportionately impacted some regions in Brazil more than others, which may be confounding our estimates, however we believe that our analysis should account for these confounders.

Anticipated health benefits of the PMM appear not to have been realised despite substantial investment in training and quality assurance including. Qualitative analysis has found qualification in the work process in the PMM, in fulfilling the work schedule and in the participation of physicians in team meetings, groups and interprofessional work (Arruda et al., 2017; Franco et al., 2019; Pereira & Pacheco, 2017). However, apparently, these changes have not had enough impact on the indicators analysed in this article.

The main contribution of this work is the estimates of the marginal treatment effect (using a continuous treatment measure). The flexibility to estimate region-specific estimates also demonstrates that there is substantial variation in the effects of increasing physician density. The work also relies on high-quality datasets in Brazil, which are routinely used for impact evaluations. One limitation includes the potential for spill-over effects, where either doctors or patients cross microregion borders to provide or access care. Other potential limitations include changes in data and recording associated with the PMM, other services changes and adaptions not measured, and substitution of existing health professionals. There are also data limitations due to extrapolated microregional characteristics and population estimates from the 2010 Census to the entire study period, and loss of granularity from aggregating data. It is also possible the generated synthetic controls (counterfactuals) are poor comparisons for each microregion, and although we present supporting evidence of the strength of the approach, there are individual cases that may violate these assumptions. The synthetic controls may also be limited due to the limited and aggregated data used in their estimation.

## Policy Implications

The results presented in this paper suggest that the PMM had limited short-term effectiveness in impacting both hospitalisations and mortality. However, the post estimation correlation analysis suggests that underserved microregions (those with low density of nurses, largest proportion of extremely poor, and rural areas) were the ones that benefit the most from receiving an additional primary care physician. Our work suggests that appropriate targeting of resources is vital for improving health outcomes and policymakers should carefully consider the appropriate allocation of these resources. The allocation of healthcare resources must also be accompanied with an understanding of how the local healthcare system will and should integrate the new doctors into the delivery of services. Our results also suggest that an increase in primary care physicians on its own is unlikely to lead to substantial changes in population health, and instead policymakers should consider increasing physician numbers as part of a larger programme of policies which strengthen the primary care system and the healthcare system overall.

## Conclusion

Using a generalised synthetic control methodology, this study finds no evidence that PMM physicians had an impact on hospitalisations or mortality on average. Policies expanding human resources for health need careful development and implementation, and consideration of health system responses and existing service arrangements. Appropriate allocation and targeting of physicians to the most vulnerable and in-need populations remains a major challenge.

## Supplementary Material

Supplementary Materials

## Figures and Tables

**Table 1:** Summary Statistics

a) Monthly Health Data		Pre vs. Post PMM Roll-out	
		Pre-PMM	Post-PMM
Total Hospitalisations per 100,000		537.545 (143.863)	534.764 (146.840)
ACSC Hospitalisations per 100,000		143.032 (70.124)	127.765 (64.722)
Total Mortality per 100,000		48.230 (13.509)	54.451 (13.683)
ACSC Mortality per 100,000		14.658 (5.546)	15.960 (5.674)
Total Primary Care Doctor Density (per 100,000)		37.944 (11.211)	41.647 (9.987)
PMM Doctor Density (per 100,000)		0.000 (0.000)	9.376 (6.742)
Non-PMM Doctor Density (per 100,000)		37.944 (11.211)	32.270 (10.957)
Health Facilities per 100,000 population		231.718 (108.913)	222.328 (94.828)
Nurses per 100,000 population		38.704 (24.150)	71.400 (39.542)
Obs.		36,894	30,154
b) 2010 Census Data			
Percentage of extremely poor	10.838 (10.549)		
Human Development Index	0.674 (0.070)		
Percentage Rural Population	5.586 (5.383)		
Population	337,855.725 (874,120.858)		
Number of Microregions	557		

Table presents summary statistics (mean and in parentheses standard deviation) of the data we use in our analysis. Panel a) includes the monthly health data separately for the pre-PMM period (before July 2013) and for the post-PMM roll-out. Panel b) includes the 2010 Census data we use. Distrito Federal not included.
