# Supplementary material for: More Doctors, better health? A generalised synthetic control approach to estimating impacts of increasing doctors under Brazil’s Mais Medicos Programme
Source: Soc Sci Med. Author manuscript; Available in PMC 2025 Sep 26. (PMC7618175; doi:10.1016/j.socscimed.2024.117222)
Supplement: Supplementary Materials [file EMS208905-supplement-Supplementary_Materials.pdf]

Supplementary material for: More Doctors, better health? Considering doctor numbers in the Mais Medicos Programme

GSC Assumptions

GSC Assumption – Synthetic Control Fit

To claim causality, key assumptions are required. Firstly, a good synthetic control for each microregion needs to be generated and needs to "look" like the microregion it is being compared to. It should evolve over time in the same way. Graphical comparison of the pre-treatment outcomes of the synthetic control and the true outcomes is necessary. We present the average Root Mean Squared Error (RMSE) of the synthetic microregion and the true outcome for that microregion, separately for each state over the period pre-PMM roll-out. Figure 1 presents these for total hospitalisations, while Figure 2 presents them for total mortality. As can be seen in these figures, there is some variation over time in the RMSE for each state, however there is no clear pattern in the RMSE. Overall, the RMSE are also relatively small (for total mortality rate it is approximately 10% of the outcome, and for hospitalisations is about 8.1% of the outcome) suggesting reasonable counter-factual are estimated for each microregion.

Figures 3 and 4 present the average of the true outcome (total mortality rate or total hospitalisation rate) and the average outcome generated by the synthetic control. Again, we present these figures separately for each state, and show these values for the entire study period (2008-2017). The red line indicates the initial PMM roll-out period. These figures show that on average the synthetic control tracks the true outcomes over the pre-period exceptionally well. Indeed, shocks and seasonality also appear to be replicated well by the synthetic control, the only exception being the spikes in hospitalisation rates in Pernambuco during 2012. There is little evidence of the synthetic control systematically under- or over-estimating the outcomes in the pre-PMM period. Overall, these figures provide strong evidence that the synthetic controls are able to generate good counterfactuals for microregions.

Figure 1 Synthetic Control Fit: Average RMSE between the true hospitalisation rate and the generated synthetic control for each State

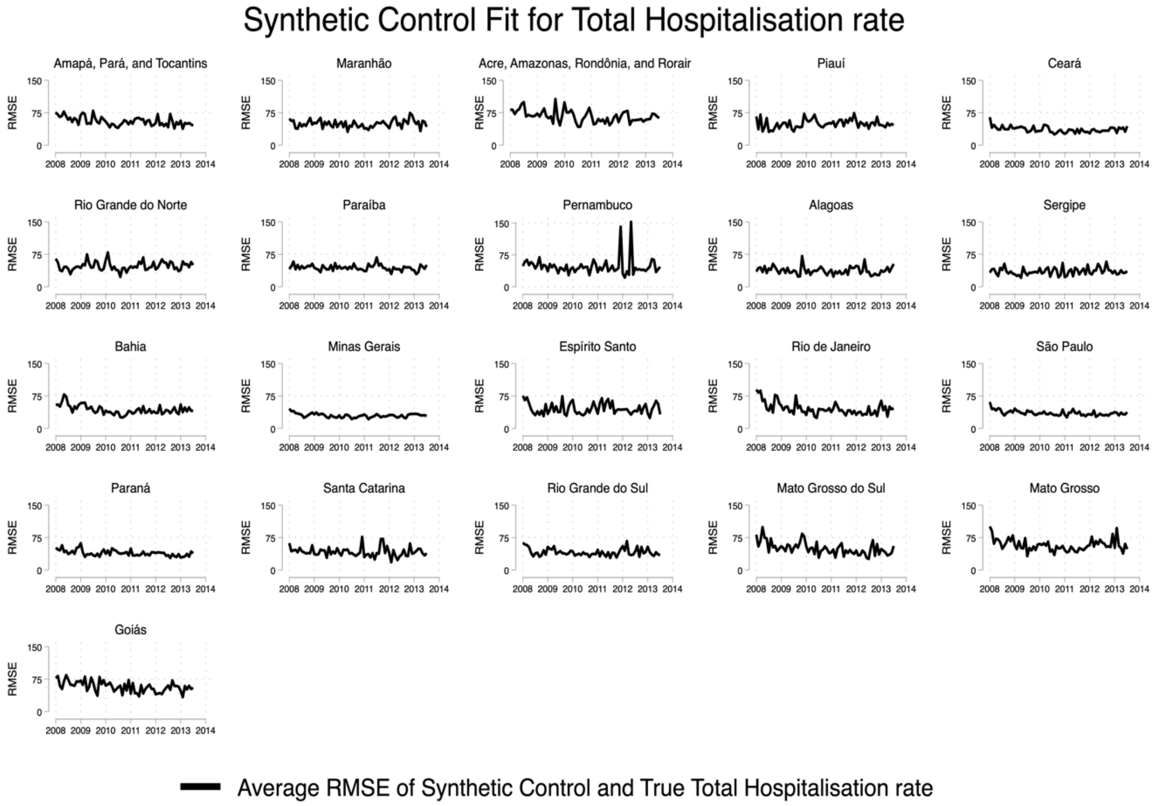

Figure shows average Root Mean Squared Error (RMSE) of the synthetic microregion and the true hospitalisation rate for that microregion separately for each. This is calculated by taking the square and then the square root of difference between the SC and the true hospitalisation rate for each microregion, and then taking an average of this value for each State. The y-axis shows the RMSE, while the x-axis are periods. The Synthetic Control is estimated synthetic control using the MM physician density independent variable specification and shows only the pre-PMM roll-out period. Distrito Federal effects are not estimated.

Figure 2 Synthetic Control Fit: Average RMSE between the true mortality rate and the generated synthetic control for each State

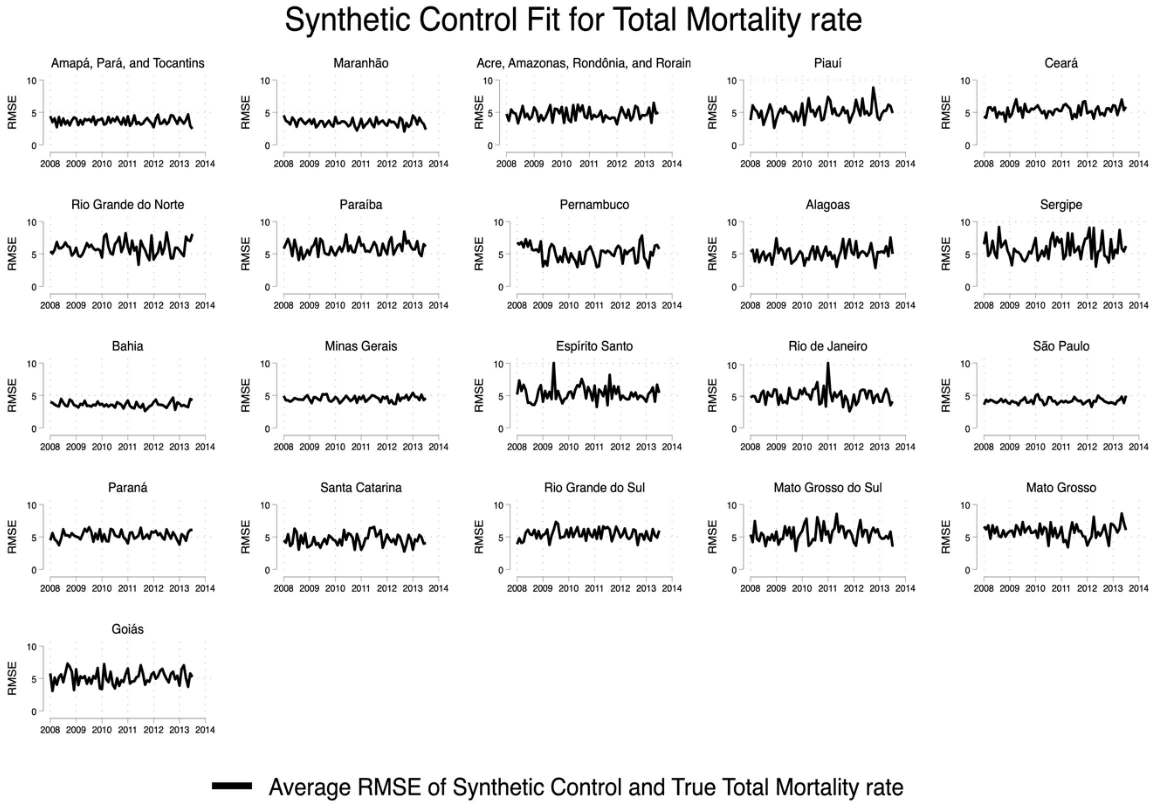

Figure shows average Root Mean Squared Error (RMSE) of the synthetic microregion and the true mortality rate for that microregion separately for each. This is calculated by taking the square and then the square root of difference between the SC and the true mortality rate for each microregion, and then taking an average of this value for each State. The y-axis shows the RMSE, while the x-axis are periods. The Synthetic Control is estimated synthetic control using the MM physician density independent variable specification and shows only the pre-PMM roll-out period. Distrito Federal effects are not estimated.

Figure 3 Synthetic Control Fit: Average hospitalisation rate and the average synthetic control hospitalisation rate for each State

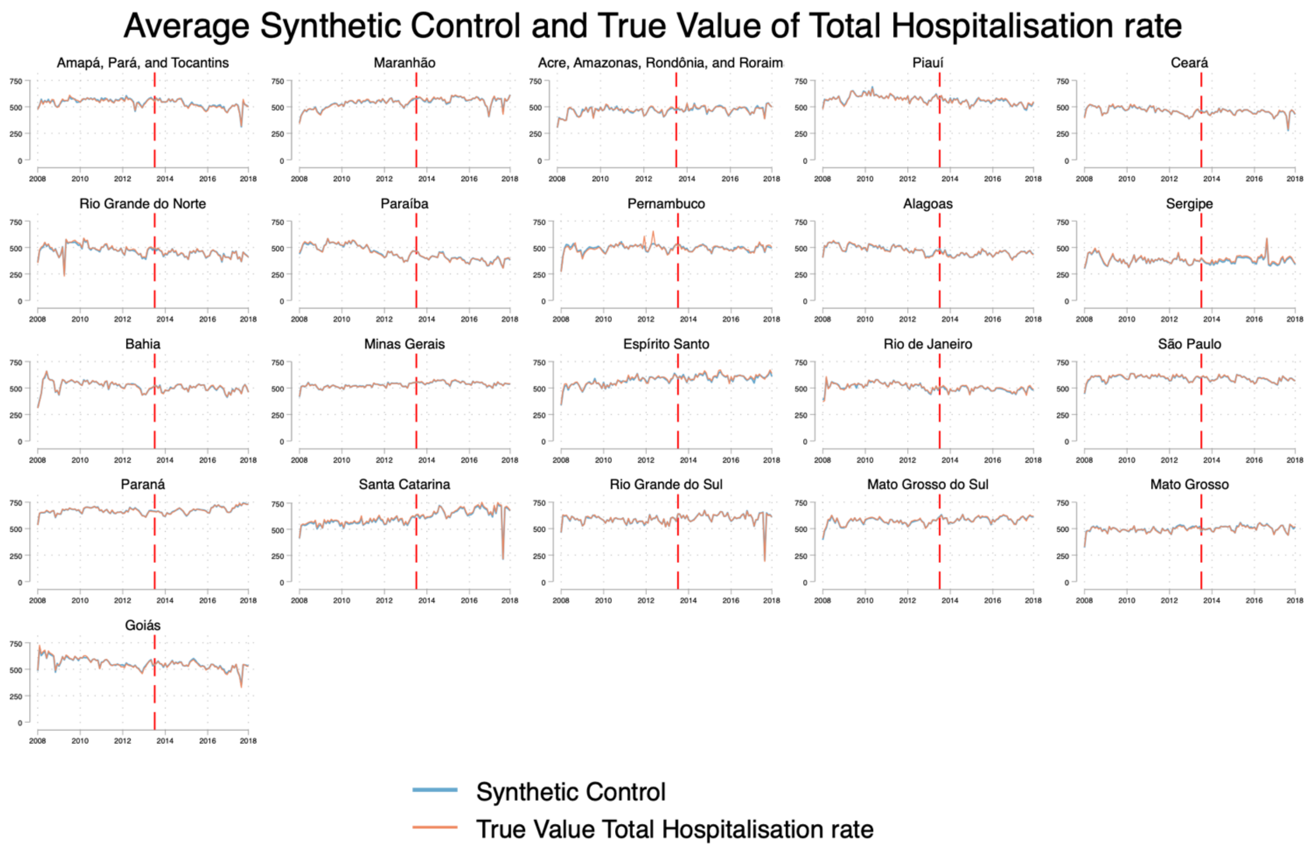

Figure shows the state average total hospitalisation rate (orange line) and the average hospitalisation rate generated by the synthetic control (blue line) for that state for the entire study period (2008-2017). The red dashed line shows the initial period of PMM roll-out. The Synthetic Control is estimated synthetic control using the PMM physician density independent variable. Distrito Federal effects are not estimated.

Figure 4 Synthetic Control Fit: Average Mortality Rate and the Average Synthetic Control Mortality Rate for Each State

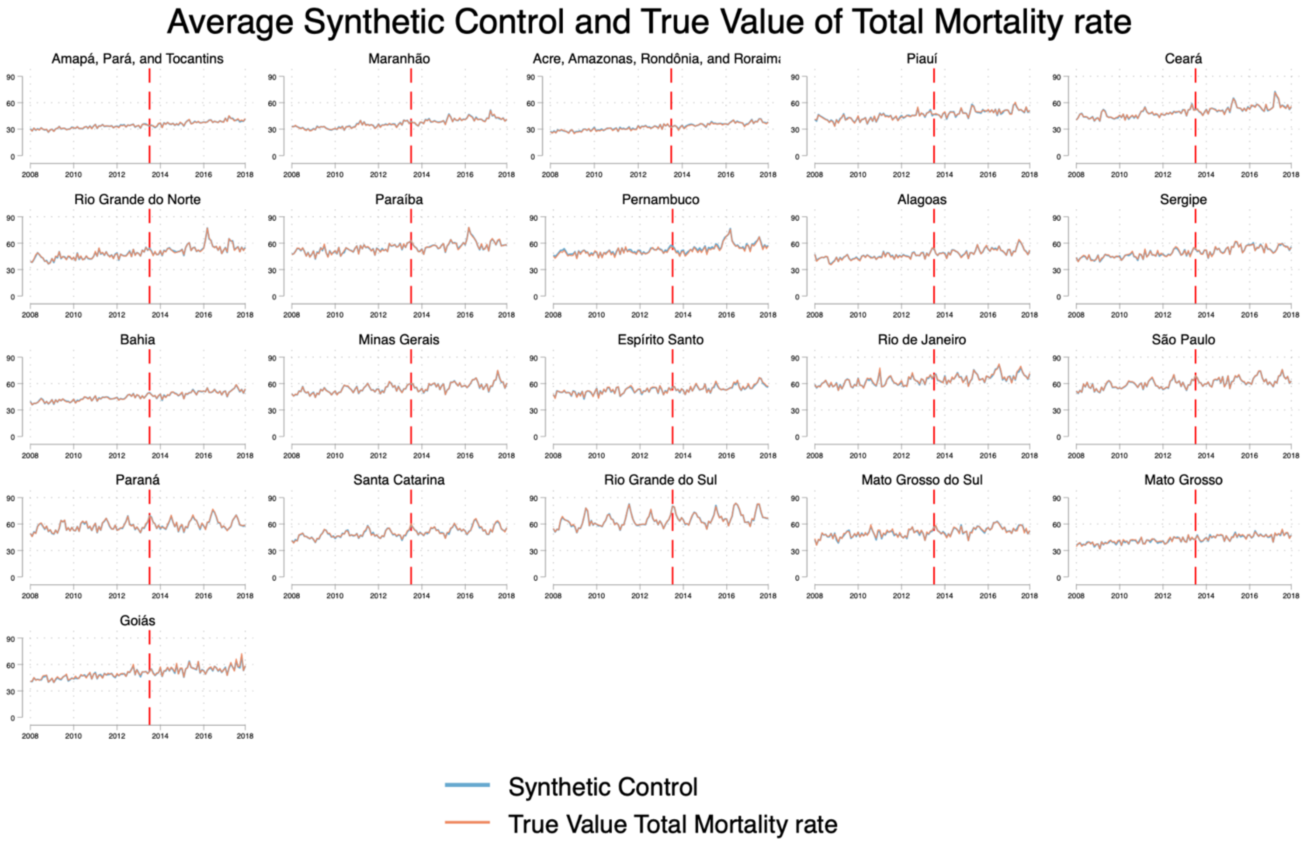

Figure shows the state average total mortality rate (orange line) and the average mortality rate generated by the synthetic control (blue line) for that state for the entire study period (2008-2017). The red dashed line shows the initial period of PMM roll-out. The Synthetic Control is estimated synthetic control using the PMM physician density independent variable. Distrito Federal effects are not estimated.

GSC Assumption – No Anticipation

An additional required assumption is that there is no anticipation of physician density, or that future physician density does not impact present values of our outcomes of interest. This is like testing pre-trends in a difference-in-differences estimator. It is difficult to imagine a situation in which microregions would be able to influence mortality in the expectation of receiving more primary care physicians in the future, however, hospitalisations could plausibly increase. Microregions could increase hospital admissions, in the knowledge that hospitals would have sufficient staff to care for these patients in the future. Although this story is unlikely to be credible, we directly test whether anticipation does exist. To test whether future physician density impacts current mortality or hospitalisations we estimate the GSC for each state, as we do in the main paper, but instead using 6-months' worth of leads for our treatment variable, which tests whether leads of physician density (i.e. future physician density numbers) have a statistically significant relationship with the outcome of interest. In favour of compactness, we don't present the p-values for each of the leads, as this would mean presenting 168 different p-values. When inspecting the models with PMM physician density as the independent variable approximately 4.7% of our pre-trends are statistically significant, and 10.7% of p-values are less than 0.1, both of which are approximately what we would expect, given the significance level and the large number of tests we conduct. For non-Mais Medicos physician models, we find that 1.2% significant at the 95% confidence level, and at the 90% confidence level approximately 3.5% of all leads are significant. Therefore, the test of these pre-trends also suggest that our assumption of no anticipation holds. Overall, of the 168 pre-trends we test, approximately 3.0% are significant at the 95% confidence level, which is below what we would expect, therefore we are confident that the GSC was able to generate good counterfactuals.

Joint Estimation of PMM and Non-PMM Physicians

In our main analysis we separately estimate the effect of PMM and non-PMM physicians on our outcomes. In this appendix section we aim to assess whether our results are robust to jointly estimating the impact of PMM and non-PMM physicians, as opposed to separately estimating these results, as we do in the main paper. Figures 5 to 8 present the results of the joint estimation for the same set of outcomes presented in the main paper by state. The light-coloured circles show the estimates when PMM and non-PMM effects are jointly estimated, while the darker shows the estimates when they are estimated separately. Blue circles correspond to the estimated effect of PMM physicians, while orange is used to represent the estimates of the effect the non-PMM physicians. Capped lines are used to represent the 95% confidence intervals, which are only available for the separately estimated effects. The 95% confidence intervals are censored at the x-axis maximum. The p-values for the joint models, which are calculated by imposing the joint restriction of PMM and non-PMM effects being equal to zero, are shown to the right of the estimates for each state.

These figures show that the estimates from the joint estimation are broadly concurrent with the estimates when the effects are estimated separately. In the majority of cases the joint and separate estimates are close to each other. Indeed, in the vast majority (approx. 85%) of cases, the joint estimates are contained within the confidence intervals for the separate estimates. Given that there is also uncertainty surrounding the joint estimates which we do not present graphically, we are confident that the estimates from the joint effect are not statistically different to those estimated separately.

This representation of the results also allows us to compare the estimated effect for PMM and non-PMM physicians. Again, the confidence intervals for PMM and non-PMM doctors overlap in majority of cases, suggesting that the effects of PMM and non-PMM physicians are not statistically different.

Figure 5 Joint Estimation of the effect of PMM and Non-PMM Physicians on Total Hospitalisations by State

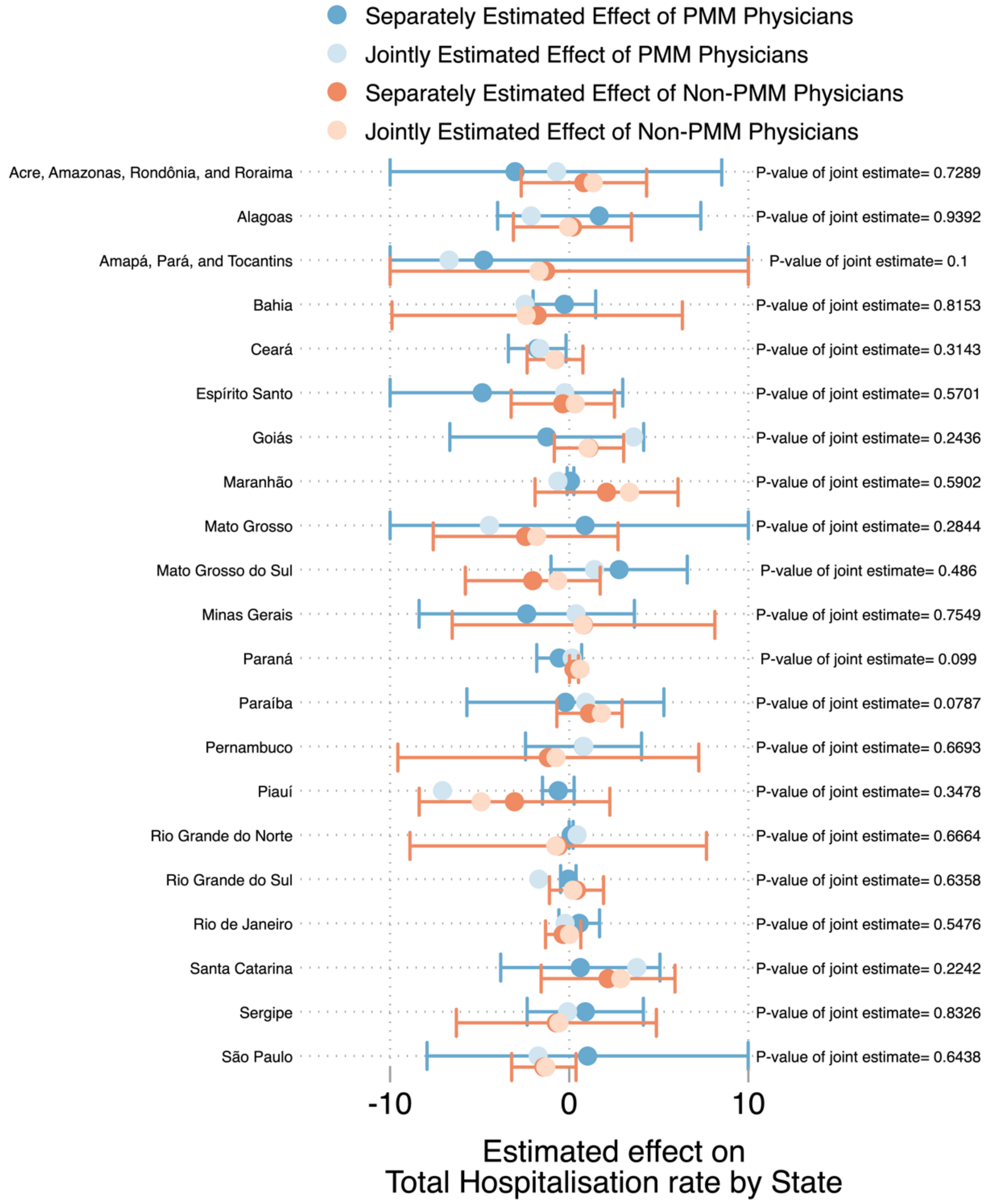

Figure presents the results of the joint and separate estimation for total hospitalisations by state. The light-coloured circles show the estimates when PMM and non-PMM effects are jointly estimated, while the darker shows the estimates when they are estimated separately. Blue circles correspond to the estimated effect of PMM physicians, while orange is used to represent the estimates of the effect the non-PMM physicians. Capped lines are used to represent the 95% confidence intervals, which are only available for the separately estimated effects. The 95% confidence intervals are censored at the x-axis maximum. The p-values for the joint models, which are calculated by imposing the joint restriction of PMM and non-PMM effects being equal to zero, are shown to the right of the estimates for each state.

Figure 6 Joint Estimation of the effect of PMM and Non-PMM Physicians on ACSC Hospitalisations by State

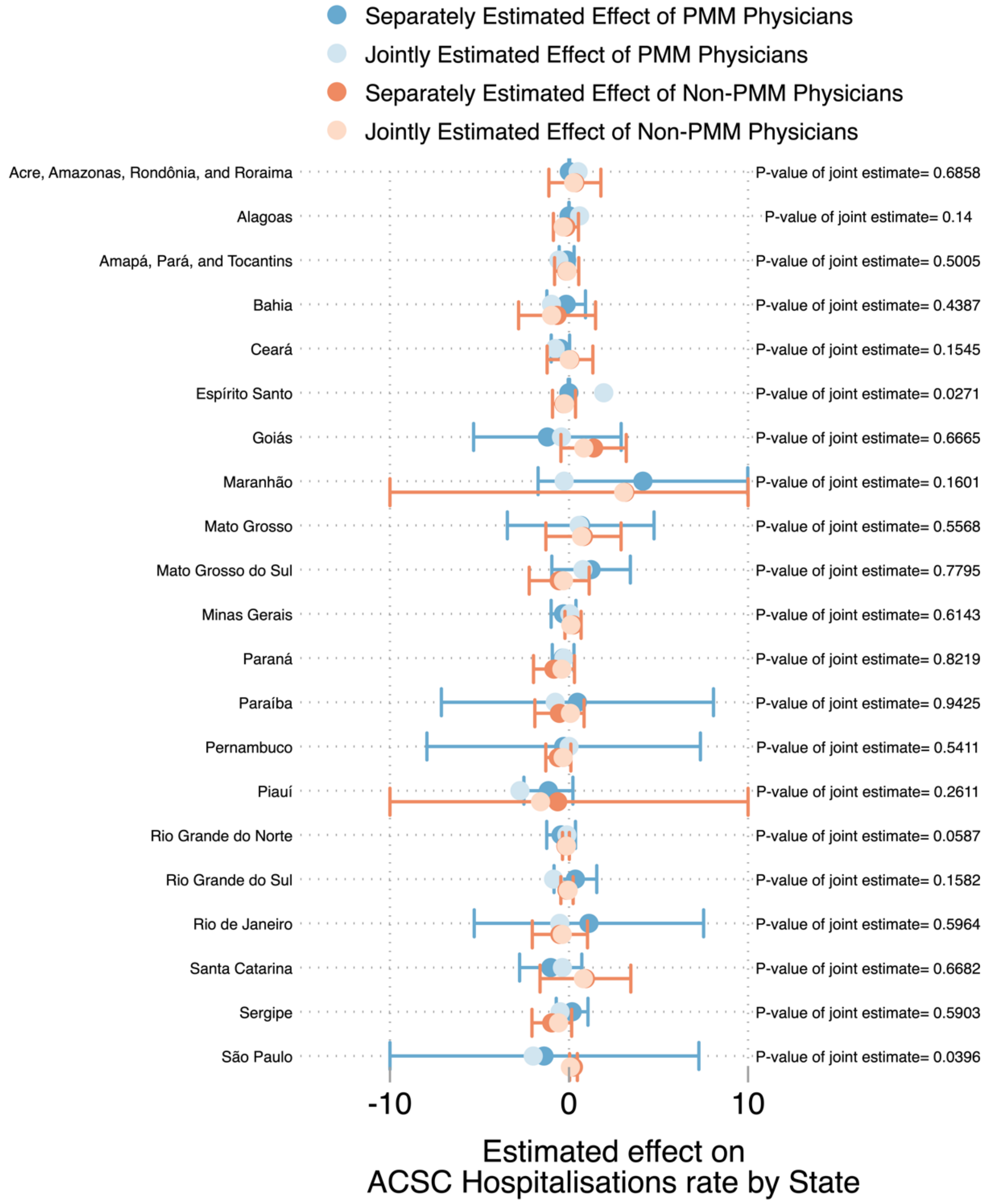

Figure presents the results of the joint and separate estimation for ACSC hospitalisations by state. The light-coloured circles show the estimates when PMM and non-PMM effects are jointly estimated, while the darker shows the estimates when they are estimated separately. Blue circles correspond to the estimated effect of PMM physicians, while orange is used to represent the estimates of the effect the non-PMM physicians. Capped lines are used to represent the 95% confidence intervals, which are only available for the separately estimated effects. The 95% confidence intervals are censored at the x-axis maximum. The p-values for the joint models, which are calculated by imposing the joint restriction of PMM and non-PMM effects being equal to zero, are shown to the right of the estimates for each state.

Figure 7 Joint Estimation of the effect of PMM and Non-PMM Physicians on Total Mortality by State

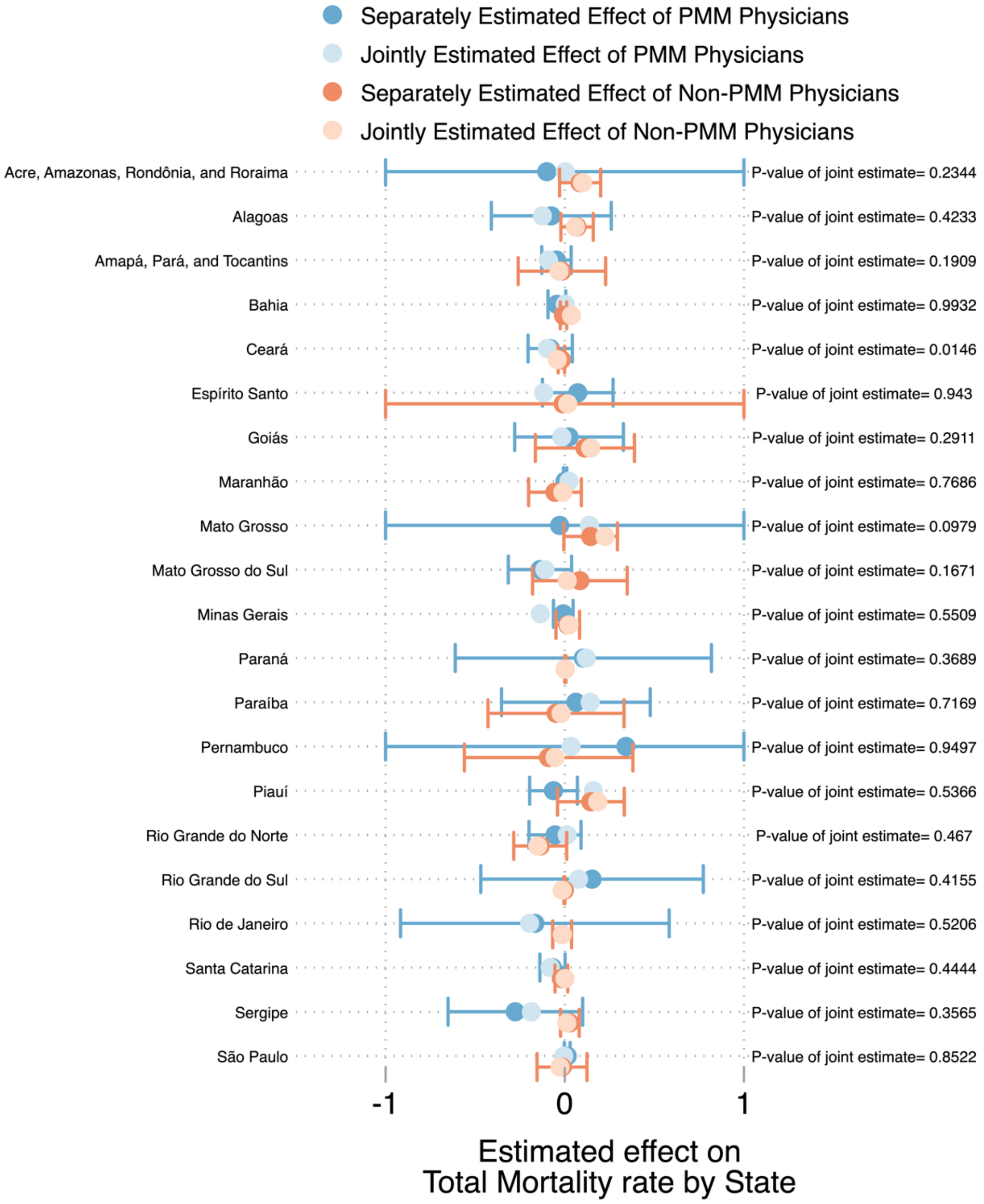

Figure presents the results of the joint and separate estimation for total mortality by state. The light-coloured circles show the estimates when PMM and non-PMM effects are jointly estimated, while the darker shows the estimates when they are estimated separately. Blue circles correspond to the estimated effect of PMM physicians, while orange is used to represent the estimates of the effect the non-PMM physicians. Capped lines are used to represent the 95% confidence intervals, which are only available for the separately estimated effects. The 95% confidence intervals are censored at the x-axis maximum. The p-values for the joint models, which are calculated by imposing the joint restriction of PMM and non-PMM effects being equal to zero, are shown to the right of the estimates for each state.

Figure 8 Joint Estimation of the effect of PMM and Non-PMM Physicians on ACSC Mortality by State

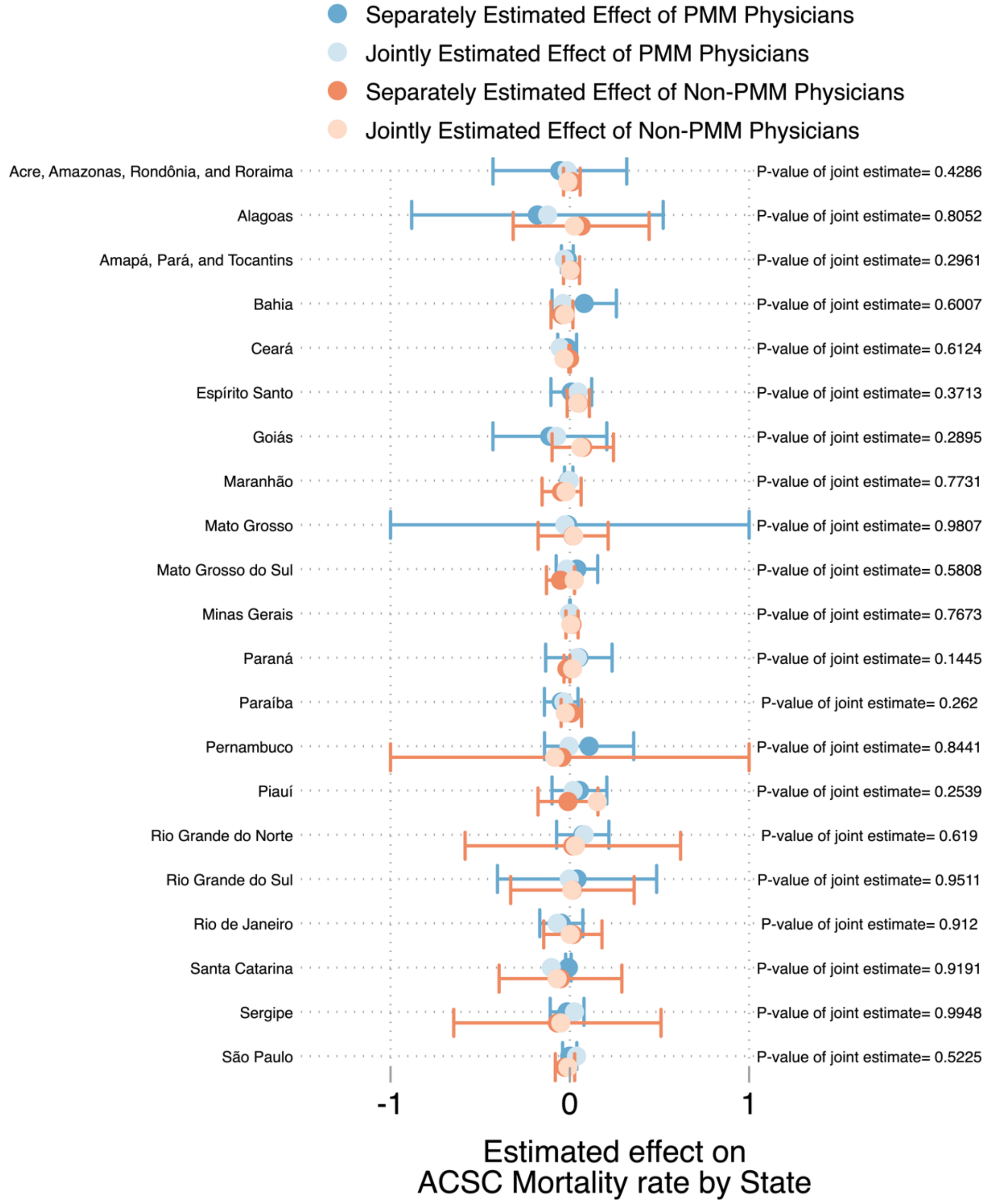

Figure presents the results of the joint and separate estimation for ACSC mortality by state. The light-coloured circles show the estimates when PMM and non-PMM effects are jointly estimated, while the darker shows the estimates when they are estimated separately. Blue circles correspond to the estimated effect of PMM physicians, while orange is used to represent the estimates of the effect the non-PMM physicians. Capped lines are used to represent the 95% confidence intervals, which are only available for the separately estimated effects. The 95% confidence intervals are censored at the x-axis maximum. The p-values for the joint models, which are calculated by imposing the joint restriction of PMM and non-PMM effects being equal to zero, are shown to the right of the estimates for each state.

Estimates of Lagged Treatment Effect

In this appendix section we present estimates of the lagged effect of PMM physicians on total hospitalisation and mortality rate. In our main analysis we use contemporaneous values of our treatment variable (number of PMM physicians) and our outcomes. However, it may not be reasonable to expect primary care physicians to have an immediate impact on levels of hospitalisation or mortality in a region, and instead we may expect effects to materialise over time. Therefore, we explore the possibility that the PMM had a delayed impact on hospitalisations and mortality.

We separately estimate models with 1-month to 6-month lag of PMM physicians (i.e. past value of PMM physicians, and current value of the outcome). These estimates are presented in Figures 9 and 10. These results show that even after 6 months the PMM physicians had limited impact on either total hospitalisation or mortality rates. In almost all models the estimates are close to zero and the 95% confidence interval contains zero. These results concur with our main estimates, which suggest that PMM physicians had limited impact on either hospitalisation or mortality rates.

Figure 9 Lagged Treatment Effect: Estimates of the effect of lagged PMM Physicians on Total Hospitalisation Rate by State

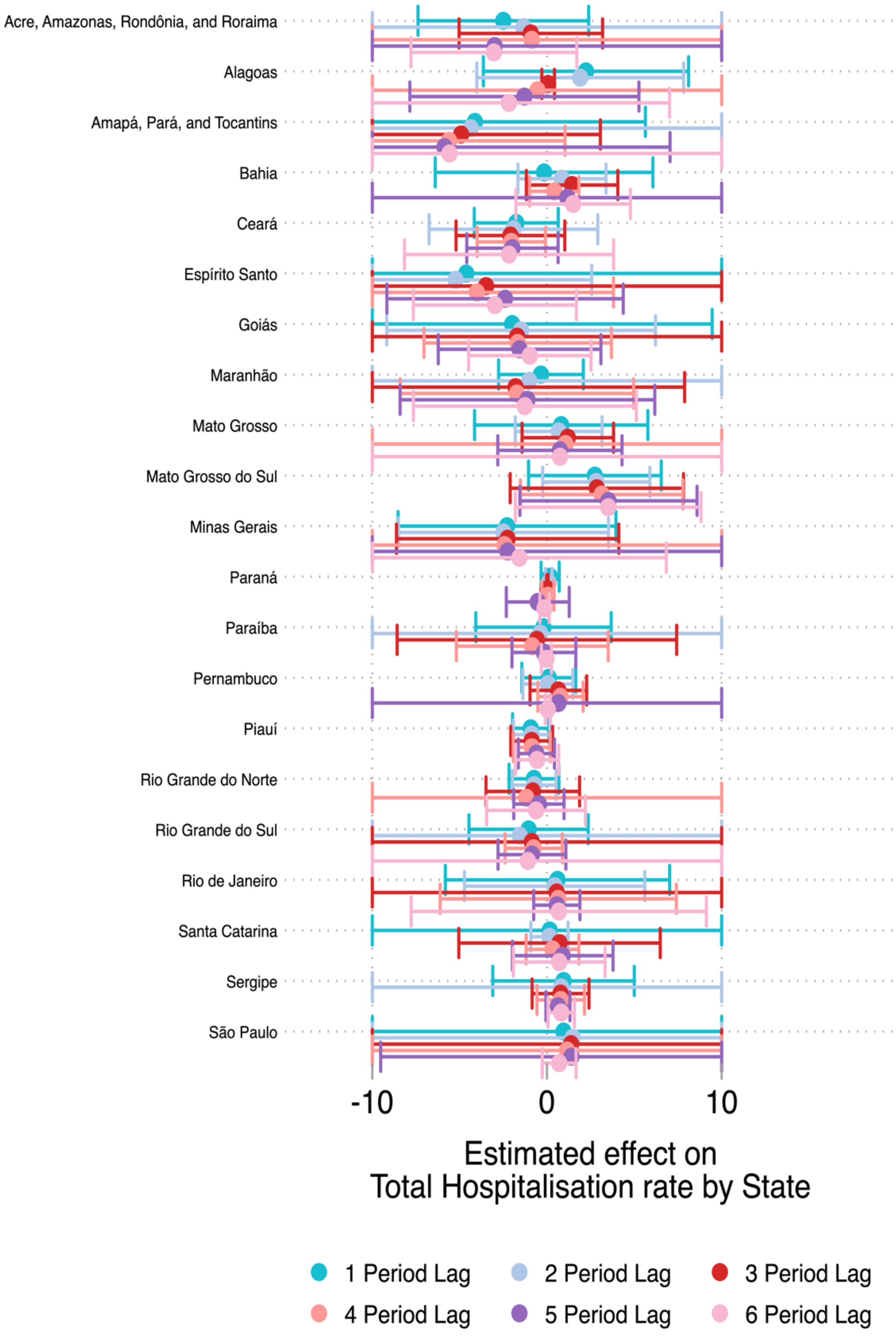

Figure presents estimates of the lagged treatment effect model on total hospitalisations for 1 to 6-months. Circles represent the estimates, and capped lines are used to represent the 95% confidence intervals. The 95% confidence intervals are censored at the x-axis maximum.

Figure 10 Lagged Treatment Effect: Estimates of the effect of lagged PMM Physicians on Total Mortality Rate by State

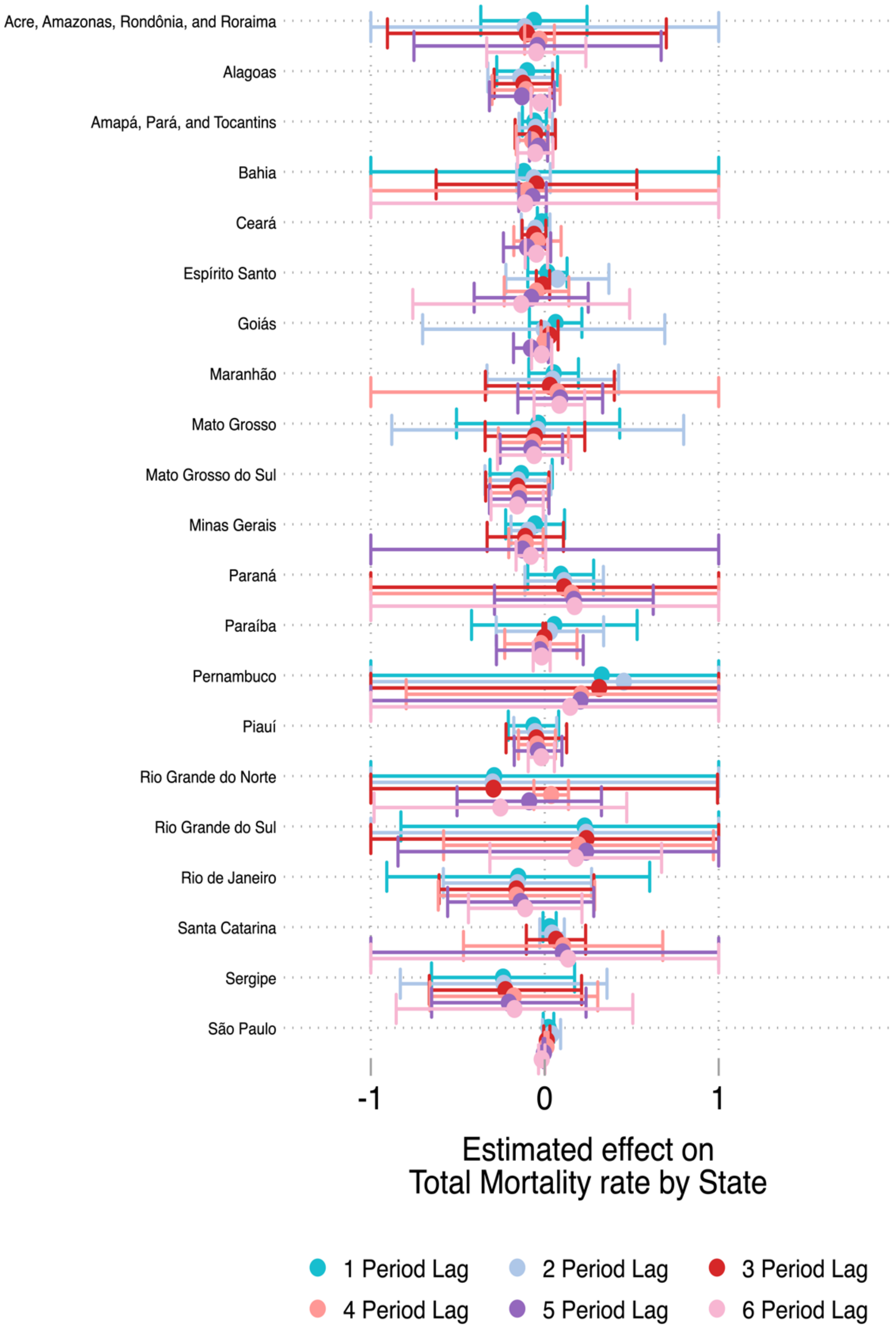

Figure presents estimates of the lagged treatment effect model on total mortality for 1 to 6-months. Circles represent the estimates, and capped lines are used to represent the 95% confidence intervals. The 95% confidence intervals are censored at the x-axis maximum.

Additional Figures and Tables

Table 1 Number and Mean Populations of Microregion and Municipalities by State

	Microregion		Municipalities	
**Federative Unit**	**Number**	**Mean Population (Standard Deviation)**	**Number**	**Mean Population (Standard Deviation)**
Acre	5	190672.7 (161617.6)	22	33343.59 (69506.91)
Alagoas	13	294672 (299117.4)	102	30593.08 (93445.98)
Amapá	4	294376.2 (260519)	16	41845.38 (98004.89)
Amazonas	13	362880.2 (606119.9)	62	56193.3 (226229.4)
Bahia	32	527200.1 (539209.6)	417	33613.68 (136041.9)
Ceará	33	358837.9 (691849.6)	184	45936.85 (182564)
Distrito Federal	1	2570160 (.)	1	2570160 (.)
Espírito Santo	13	270292.6 (351490.7)	78	45063.49 (83759.2)
Goiás	18	420712 (535422.6)	246	24405.64 (92651.86)
Maranhão	21	345376.6 (224040.9)	217	30298.57 (73225.47)
Mato Grosso	22	142419.4 (148431.7)	141	21525.69 (54105.74)
Mato Grosso do Sul	11	283645.4 (248714.9)	79	31397.74 (90848.02)
Minas Gerais	66	390346.8 (763452.3)	853	22974.6 (93751.55)
Paraná	39	357537.9 (619591.2)	399	26176.76 (98378.51)
Paraíba	23	155072.1 (167592.8)	223	16890.26 (55804.33)
Pará	22	364890.2 (386561.2)	144	53014.34 (125234.8)
Pernambuco	19	514754.7 (617119.4)	185	47548.37 (129168.6)
Piauí	15	235071.1 (215561.3)	224	13921.25 (55469.2)
Rio Grande do Norte	19	143190.9 (141220.4)	167	18970.22 (66956.21)
Rio Grande do Sul	35	382331.3 (713746.7)	497	21560.34 (75794.98)
Rio de Janeiro	18	2265082 (4311524)	92	173803.6 (672081)
Rondônia	8	227699.7 (149266.6)	52	30046.33 (60996.29)
Roraima	4	117335.3 (126836.8)	15	30031.93 (70515.54)
Santa Catarina	20	326093.9 (208243)	295	21325.72 (50687.11)
Sergipe	13	146018.6 (169473)	75	27573.56 (68161.06)
São Paulo	63	648517.9 (1557795)	645	63972.4 (454386.5)
Tocantins	8	168945.5 (73451.8)	139	9952.842 (24098.65)

Figure 11 Mais Medicos and Non-Mais Medicos Physician Density by Microregion

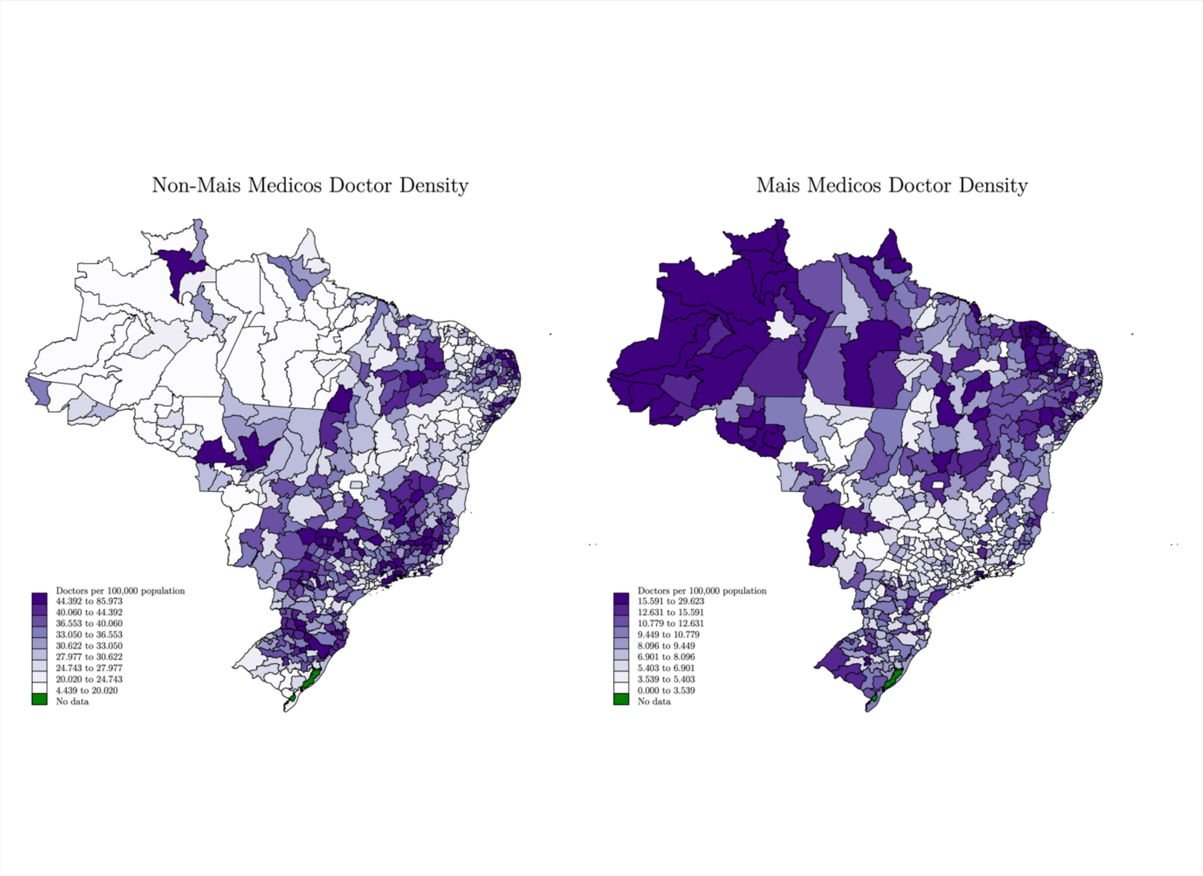

Figure 12 Number of Primary Care Physicians in Brazil

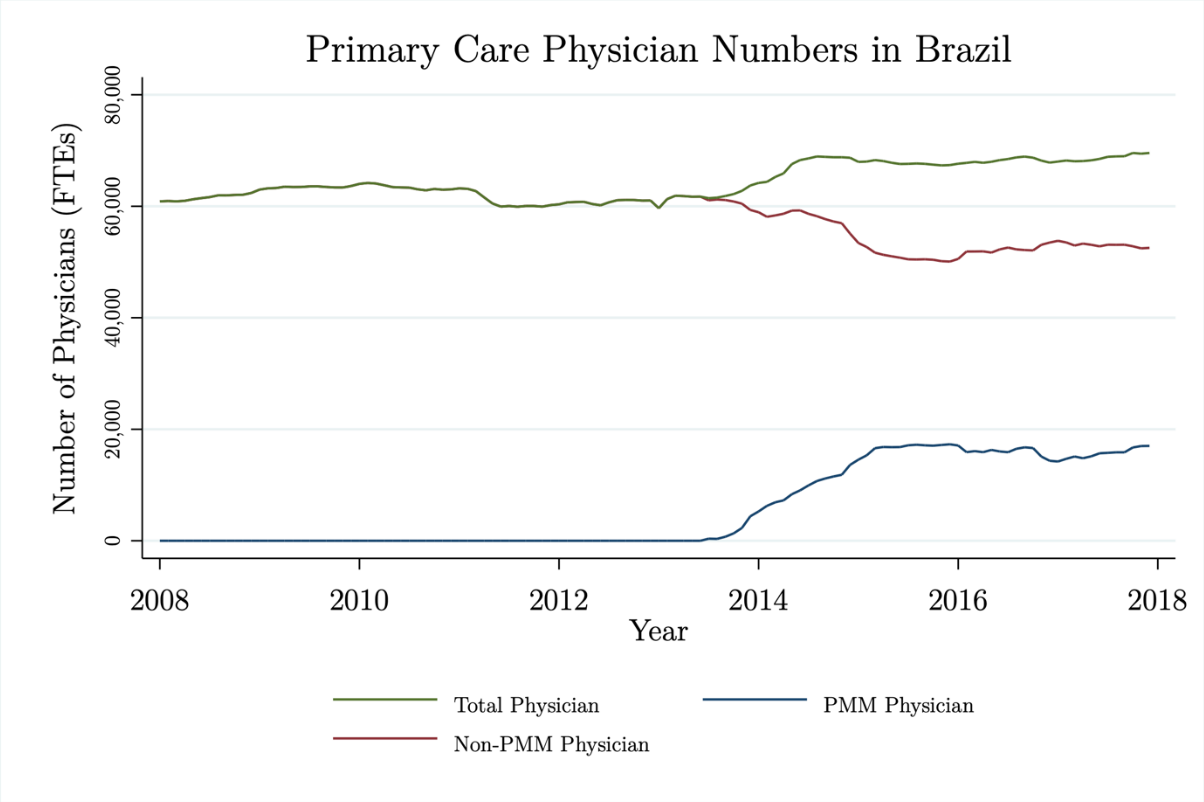

Figure 13 Primary Care Physicians Density by State

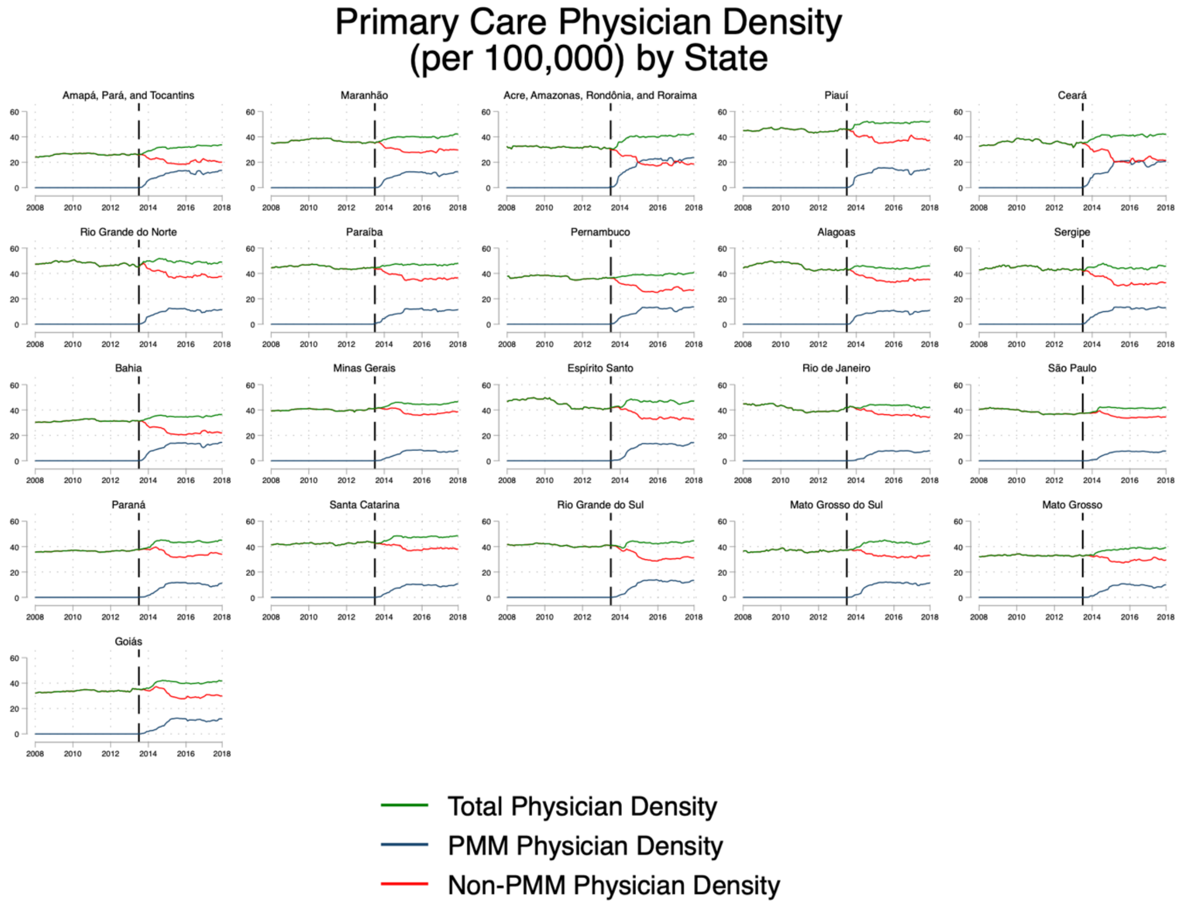

Figure 14 Hospitalisations per month-year

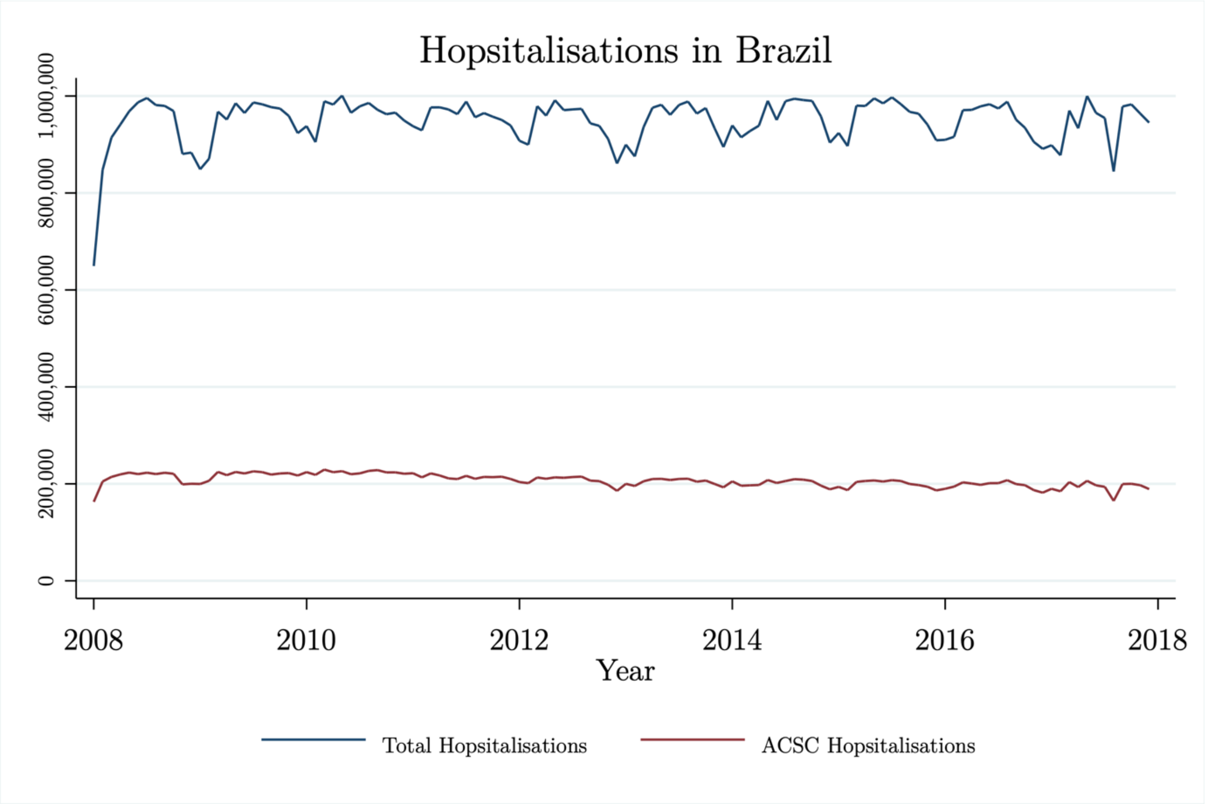

Figure 15 Mortality per month-year

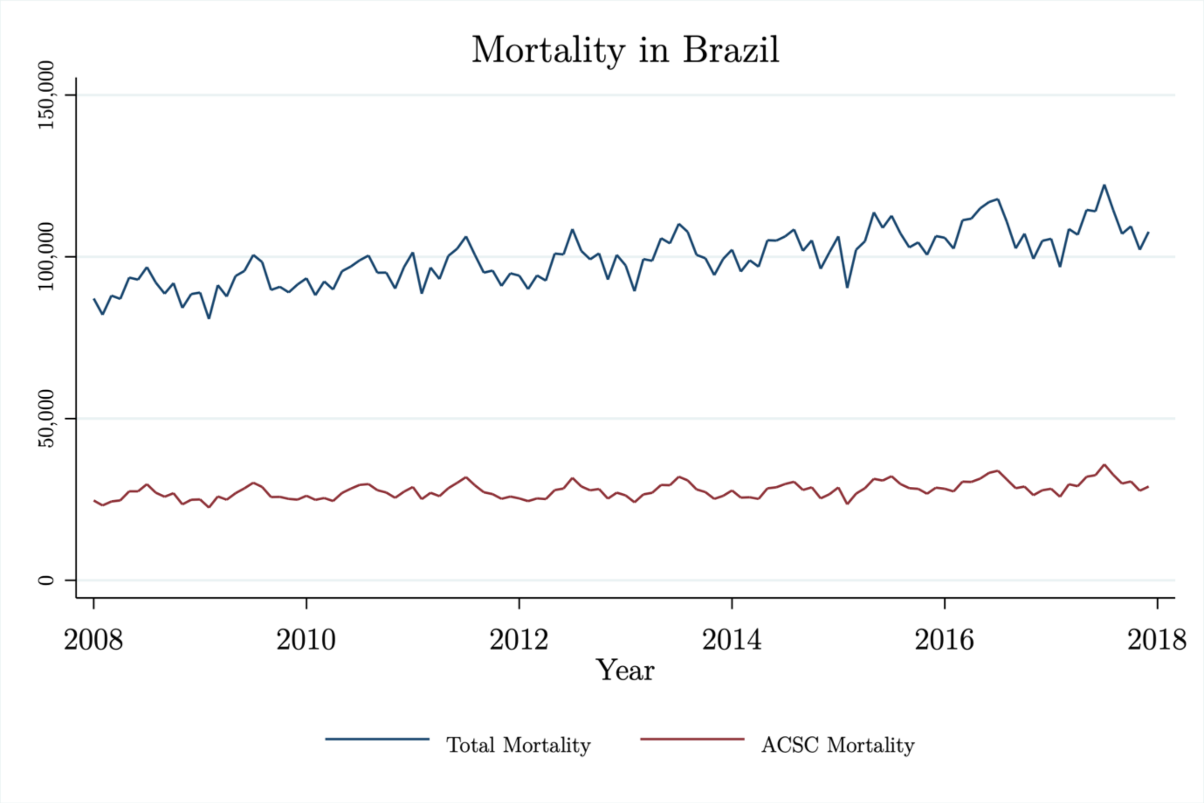

Figure 16 Effect of an increase in PMM physician density on Total and ACSC Hospitalisation rate by Microregion

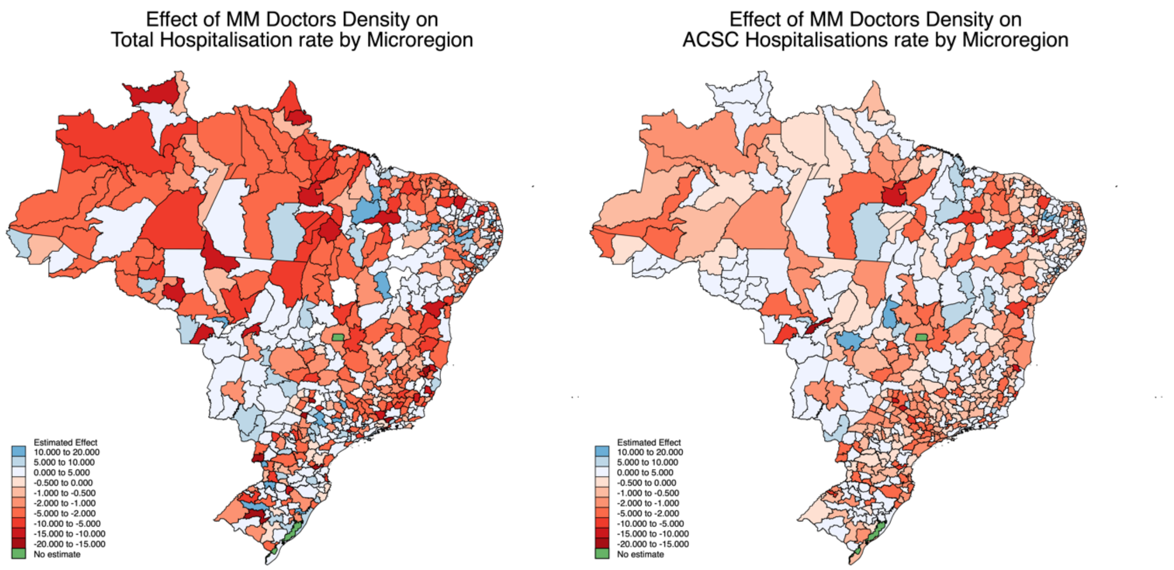

Microregion-Specific GSC Estimates of the effect of a unit increase in PMM *physician density (per 100,000 population) on the Hospitalisation rate (Total or ACSC, per 100,00 population) shown graphically. Blue represents values above zero, whereas red represents negative values, shade of each represents the magnitude. Estimates are shown for descriptive purposes, and statistical inference is not conduced on these estimates. Distrito Federal estimates are not included.*

Figure 17 Effect of an increase in non-MM physician density on Total and ACSC Hospitalisation rate by Microregion

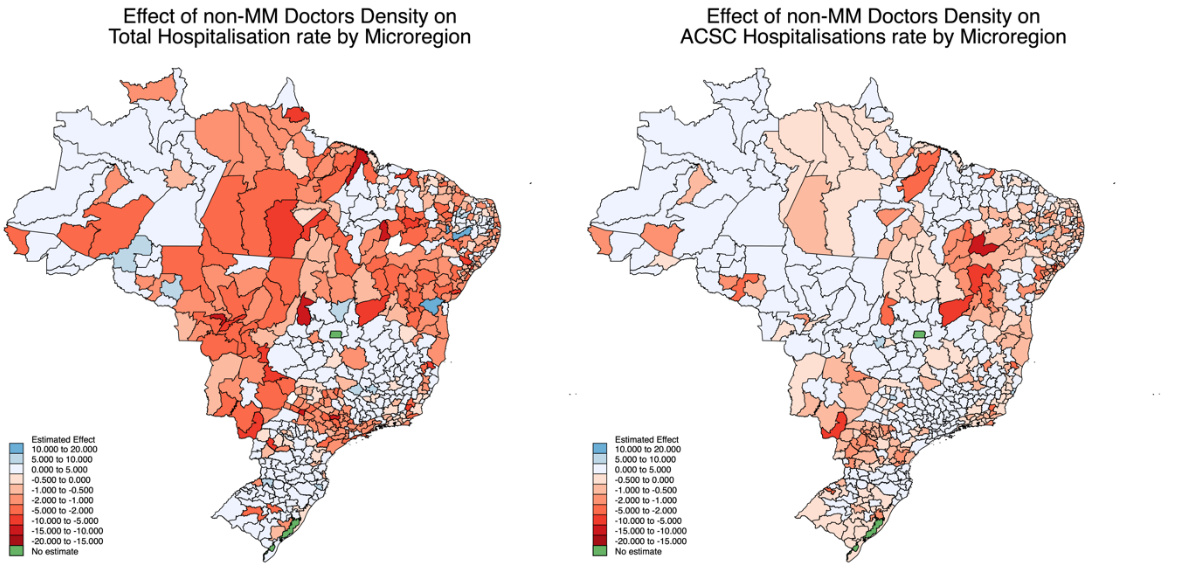

*Microregion-Specific GSC Estimates of the effect of a unit increase in non-*PMM *physician density (per 100,000 population) on the Hospitalisation rate (Total or ACSC, per 100,00 population) shown graphically. Blue represents values above zero, whereas red represents negative values, shade of each represents the magnitude. Estimates are shown for descriptive purposes, and statistical inference is not conduced on these estimates. Distrito Federal estimates are not included.*

Figure 18 Effect of an increase in PMM physician density on Total and ACSC Mortality rate by microregion

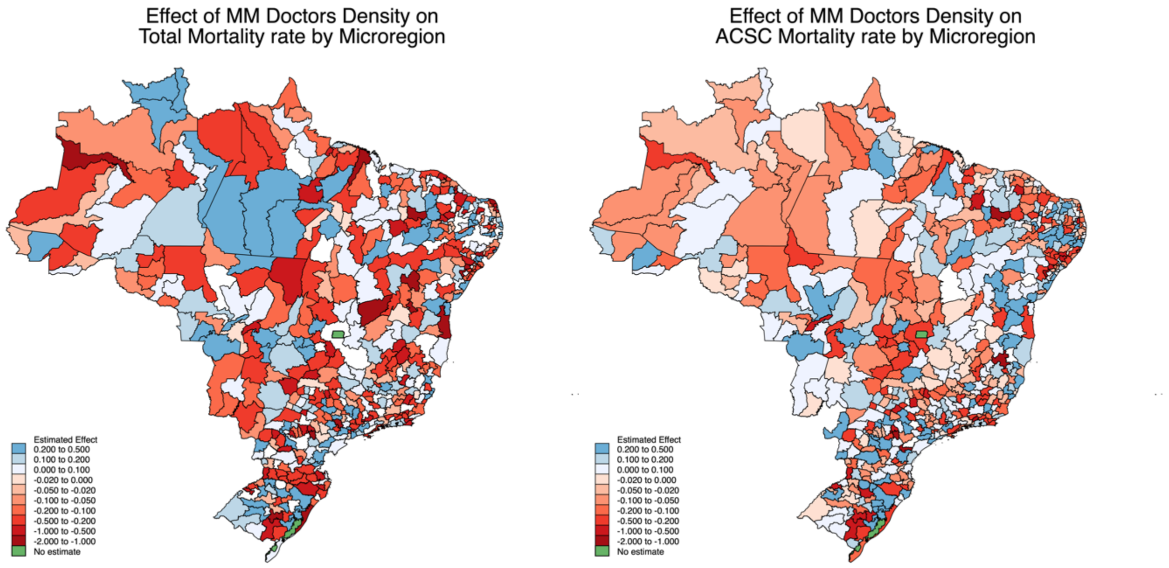

Microregion-Specific GSC Estimates of the effect of a unit increase in PMM *physician density (per 100,000 population) on the Mortality rate (Total or ACSC, per 100,00 population) shown graphically. Blue represents values above zero, whereas red represents negative values, shade of each represents the magnitude. Estimates are shown for descriptive purposes, and statistical inference is not conduced on these estimates. Distrito Federal estimates are not included.*

Figure 19 Effect of an increase in non-MM physician density on Total and ACSC Mortality rate by microregion

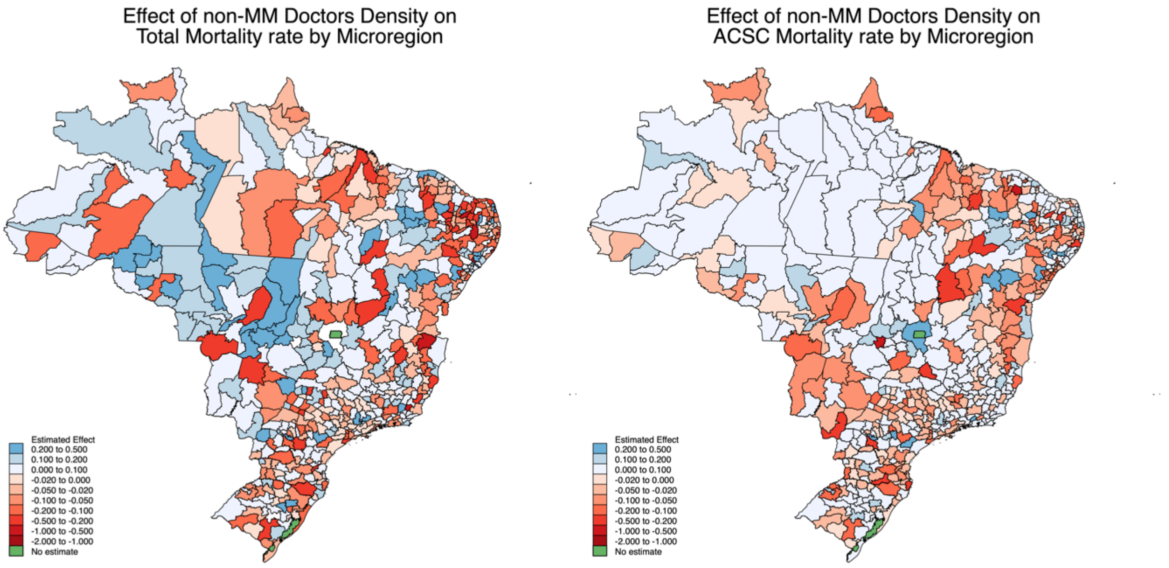

*Microregion-Specific GSC Estimates of the effect of a unit increase in non-*PMM *physician density (per 100,000 population) on the Mortality rate (Total or ACSC, per 100,00 population) shown graphically. Blue represents values above zero, whereas red represents negative values, shade of each represents the magnitude. Estimates are shown for descriptive purposes, and statistical inference is not conduced on these estimates. Distrito Federal estimates are not included.*
